# Clustering of Sex-Biased Genes and Transposable Elements in the Genome of the Medaka Fish *Oryzias latipes*

**DOI:** 10.1093/gbe/evab230

**Published:** 2021-10-08

**Authors:** Corentin Dechaud, Sho Miyake, Anabel Martinez-Bengochea, Manfred Schartl, Jean-Nicolas Volff, Magali Naville

**Affiliations:** 1 Institut de Genomique Fonctionnelle de Lyon, Univ Lyon, CNRS UMR 5242, Ecole Normale Superieure de Lyon, Universite Claude Bernard Lyon 1, Lyon, France; 2 Entwicklungsbiochemie, Biozentrum, Universität Würzburg, Würzburg, Germany; 3 Department of Chemistry and Biochemistry, The Xiphophorus Genetic Stock Center, Texas State University, San Marcos, Texas, USA

**Keywords:** Transposable elements, gonads, fish, genome organization, RNA-seq

## Abstract

Although genes with similar expression patterns are sometimes found in the same genomic regions, almost nothing is known about the relative organization in genomes of genes and transposable elements (TEs), which might influence each other at the regulatory level. In this study, we used transcriptomic data from male and female gonads of the Japanese medaka *Oryzias latipes* to define sexually biased genes and TEs and analyze their relative genomic localization. We identified 20,588 genes expressed in the adult gonads of *O. latipes*. Around 39% of these genes are differentially expressed between male and female gonads. We further analyzed the expression of TEs using the program SQuIRE and showed that more TE copies are overexpressed in testis than in ovaries (36% vs. 10%, respectively). We then developed a method to detect genomic regions enriched in testis- or ovary-biased genes. This revealed that sex-biased genes and TEs are not randomly distributed in the genome and a part of them form clusters with the same expression bias. We also found a correlation of expression between TE copies and their closest genes, which increases with decreasing intervening distance. Such a genomic organization suggests either that TEs hijack the regulatory sequences of neighboring sexual genes, allowing their expression in germ line cells and consequently new insertions to be transmitted to the next generation, or that TEs are involved in the regulation of sexual genes, and might therefore through their mobility participate in the rewiring of sex regulatory networks.

Significance

We analyzed RNA-seq data from medaka fish gonads, and estimated gene and transposable element (TE) expression. We report that sex-biased genes in *Oryzias latipes* gonads are arranged in clusters in the genome along with TEs. As germline expression of TEs is essential for their transmission to the next generation, we investigate the different mechanisms leading to this expression, either by being coexpressed with sexual genes, or by bringing their own regulatory sequences that will coregulate surrounding genes. Our findings are in favor of the second hypothesis. We also show that our method can discover particular TE families that are good candidates to control gene expression.

## Introduction

With ∼26,000 species (Nelson et al. 2016), teleost fish form the largest group of extant vertebrates. They present a high diversity of morphology, physiology, and behavior, this diversity also affecting their sexual development and function (Volff et al. 2007; Kobayashi et al. 2013). Many sexual modes exist in fish, from hermaphroditism, where one individual can have both sexes, either simultaneously or sequentially, to gonochorism (individuals are either male or female). Sex determination, corresponding to the process by which the future sex is decided in gonochoristic species, is also diverse in teleosts. Several systems exist, ranging from environmental sex determination (where sex can be determined by water temperature for example) to genetic sex determination (GSD, where sex is controlled by a particular gene or a particular set of genes) with or without sex chromosomes (Bachtrog et al. 2014). In some fish species, both environmental and GSD occur and interact. In the medaka fish *O.latipes*, for instance, where sex is controlled by an XY chromosome system, high temperatures trigger female-to-male sex reversal (Adolfi et al. 2019). In mammals, the mechanism of GSD is highly conserved and ancient (∼180–210 Ma; Waters et al. 2007), with almost all mammals using *Sry* as the male master sex-determining gene present on the Y chromosome. In contrast, in fish with GSD, different master sex determination genes can exist in different species, and in most species the master gene is still unknown (Kikuchi and Hamaguchi 2013). In *O. latipes* and in its related species *Oryziascurvinotus*, sex is controlled by the *dmrt1by* gene located on the Y chromosome. The *dmrt1by* gene appeared ∼10 Ma in the common ancestor of *O. curvinotus* and *O. latipes* and was subsequently lost in *Oryziasluzonensis*, the sister species of *O. curvinotus* (Kikuchi and Hamaguchi 2013). In *O. luzonensis*, the master sex-determining gene is *Gsdf*, coding for the gonadal soma-derived factor. This gene is located on both X and Y chromosomes but in two different allelic forms, with the *Gsdf^Y^* allele triggering male differentiation. In *Oryzias dancena*, the master sex*-*determining gene is *Sox3Y* that evolved from *Sox3* as *Sry* did in mammals (Takehana et al. 2014; Herpin and Schartl 2015). The *Oryzias* group thus illustrates the high variability of master sex-determining genes that can control GSD in fish.

Sexual genes can be involved in sex determination (i.e., when sex is defined), but also in sexual differentiation (i.e., when the undifferentiated gonads become testes or ovaries) or sexual function. A way to detect such genes is to analyze gene expression between males and females, usually in the gonads, but not only (Grath and Parsch 2016), and to retrieve differentially expressed genes, called sex-biased genes, that is genes more expressed in males than in females or vice versa. An evolutionary conserved feature of sex-biased genes is their fast evolution, due to stronger positive selection, as observed in Drosophila (Assis et al. 2012), *Caenorhabditiselegans* (Cutter and Ward 2005), fish (Yang et al. 2016), and primates (Khaitovich et al. 2005), showing that this trend is conserved throughout evolution. Moreover, sex-biased genes often appear not randomly distributed on chromosomes. In mice and flies, female-biased genes are preferentially located on the X chromosome (Meisel et al. 2012). In Drosophila and mouse, testis-biased genes tend to co-localize in the genome and form clusters (Boutanaev et al. 2002; Li et al. 2005; Dorus et al. 2006).

Teleost fish genomes also harbor a large diversity of transposable element (TE) families compared with other vertebrates, particularly birds and mammals (Chalopin et al. 2015). TEs are sequences able to insert in the genome. They are often repeated and found in the genome of all eukaryotes analyzed to date. The high TE diversity observed in fish constitutes an important source of potential regulatory motifs for host genes. Indeed, if the majority of TE insertions are neutral or deleterious for the host, some can also be selected for adaptive functions. Several examples have been described of TEs with major roles in the rewiring of gene regulatory networks (Feschotte 2008; Lynch et al. 2011; Rebollo et al. 2012; Sorrells and Johnson 2015; Chuong et al. 2017), some of which are related to sex (Herpin et al. 2010; Ellison and Bachtrog 2013; Ellison and Bachtrog 2015; Dechaud et al. 2019). Interestingly, the expression of *dmrt1by*, the master sex-determining gene of *O. latipes*, is partly controlled by a regulatory sequence carried by a TE called *Izanagi* (Herpin et al. 2010). This TE-derived enhancer allows the tightly regulated expression of *dmrt1by* limited to a short period of time before hatching, when sex determination occurs.

TEs are not randomly distributed in the genome. Patterns of TE insertions result from insertion preferences, selection, and genetic drift (Bourque et al. 2018). Because only TEs that insert in germline cells can be fixed in the genome to be transmitted to the next generation, these patterns could be particularly influenced by the structure of the chromatin in these cells, and thus related to gene expression. Conversely, TEs could bring regulatory elements with them and modify the expression of neighboring genes, participating in the evolution of regulatory networks in germ cells but also in gonads in general. As a first step to disentangle these potential functional regulatory links between sexual genes and TEs, we investigate here the localization of sex-biased TEs with respect to sex-biased genes. As sexual development is particularly diverse in *Oryzias* and TEs are highly diverse in teleost fish, we decided to use *O.latipes* as a model of study. We generated RNA-seq data from male and female adult gonads of *O. latipes*, and identified genes and TEs with sex-biased expression. Although gene expression in the genome is equivalently biased between males and females, TEs globally present a clear male-biased expression in gonads. We show that the closer the genes and TEs are, the more similar is their expression bias. Additionally, TEs located in sex-biased gene clusters tend to follow the cluster expression bias. Finally, we find that some male-biased TE families are enriched in male-biased gene clusters. These families constitute good candidates for TEs potentially involved in sexual gene regulation and its variability. Altogether, our study constitutes a first step toward a better understanding of the mutual regulatory influence between genes and TEs in the gonads.

## Results

### Identification of Gonadal Sex-Biased Genes

We first identified sex-biased genes by sequencing the transcriptome of three testis and three ovary replicates of *O. latipes*. These gonadal tissues are composed of both germline and somatic cells, as the two populations cannot be simply separated by dissection. However, as we are interested here in sexual function in general, and not only in germ cells, this is not limiting for our study. In addition, this did not prevent identification of sex-biased germ cell genes, for instance genes expressed in spermatogenesis (see below). Because gonads are specialized tissues that were not used to construct the reference genome annotation, this annotation (25,167 protein coding and noncoding genes) could lack some transcripts expressed in our data. To take this into account, we decided to apply the “*new tuxedo*” approach (Pertea et al. 2016), which is based on the reconstruction of a transcript annotation from the coordinates of the read alignments on the genome and the comparison of this new annotation with the reference from refseq (NCBI reference ASM223467v1; see Materials and Methods). We detected 45,444 expressed transcripts corresponding to 27,096 genes according to the pipeline. These gene models contain protein coding genes, noncoding genes, miss-assembled transcripts due to bioinformatic predictions, TEs, or any other type of expressed polyA RNA. We filtered these gene models to generate a set of coding genes and a set of noncoding genes. Among the 17,254 coding genes detected, 16,586 were already present in the refseq annotation (96.1%), and among the 3,334 noncoding genes detected, 1,845 were already present in the refseq annotation (55.3%).

On the whole, 40% of coding genes were found to be sex-biased, including 3,600 (20.9%) genes overexpressed in testis compared with ovary, and 3,293 (19.1%) genes overexpressed in ovary compared with testis. The remaining 10,361 genes were not differentially expressed (supplementary fig. 1, Supplementary Material online). The coding transcriptome is thus equivalently biased between testis and ovary. For what follows, we define genes differentially expressed between male and female gonads as “sex-biased genes.” We assume that they could be involved in sexual differentiation, maintenance, or function.

With respect to the expression of particular genes, we compared the patterns we obtained with previous studies drawn in medaka only. Indeed, recent observations suggest that sexual gene expression might vary greatly between species, which would reflect the rapid evolution of this pathway (Herpin and Schartl 2015). Some studies analyzed the expression of few genes in the gonads of *O. latipes* by RT-qPCR (Nakamoto et al. 2006; Herpin et al. 2013; Horie et al. 2016; Kobayashi et al. 2017). The main genes that have been studied in detail are: *dmrt1*, the ancestral paralog of *dmrt1by*; *gsdf*, the gonadal soma-derived factor; and *foxl2*, a transcription factor involved in ovarian development. Both *dmrt1* and *gsdf* are involved in testis development. *dmrt1*, along with *gsdf*, was always found to be overexpressed in testis compared with ovary, which corresponds to our data (supplementary fig. 1, Supplementary Material online). *foxl2* was found to be highly expressed in ovary in previous studies, and is coherently detected as ovary-biased using our RNAseq data (supplementary fig. 1, Supplementary Material online). Additionally, reverse transcriptase–polymerase chain reaction (RT-PCR) experiments detected an expression of *amh* and *sox9b* in both gonads, and of *aromatase* in ovary only (Kurokawa et al. 2007). We observe similar patterns of expression for these genes using RNAseq data.

In the case of noncoding genes, 33.2% were found to be sex-biased, including 695 (20.8%) genes overexpressed in testis, and 412 (12.4%) genes overexpressed in ovary compared with testis. The noncoding transcriptome is thus more male-biased than the coding transcriptome, with a lower contribution of ovary-biased genes.

### Identification of TE Copies and Families with Sex-Biased Gonadal Expression

To analyze TE expression relative to gene expression, we then characterized gonadal expression of TEs at the single copy level. Our annotation of TEs (supplementary data 1 and 2, Supplementary Material online) covers 34% of the medaka genome, which corresponds to a previously obtained coverage (Chalopin et al. 2015). SQuIRE (Yang et al. 2019) allows retrieval of the expression of each TE locus from RNAseq data. We identified 37,108 loci as expressed TE copies (corresponding to 3.7% of all TE loci). Among them, 13,325 (35.9%) were found to be testis-biased (adjusted *P* value<0.05, log_2_ fold-change (log_2_FC)<–1), whereas 3,842 (10.4%) were ovary-biased (adjusted *P* value<0.05, log_2_FC>1). Therefore, whereas the same proportion of coding genes was found to be testis- or ovary-biased, TE expression appears clearly biased toward testis.

**Fig. 1.**
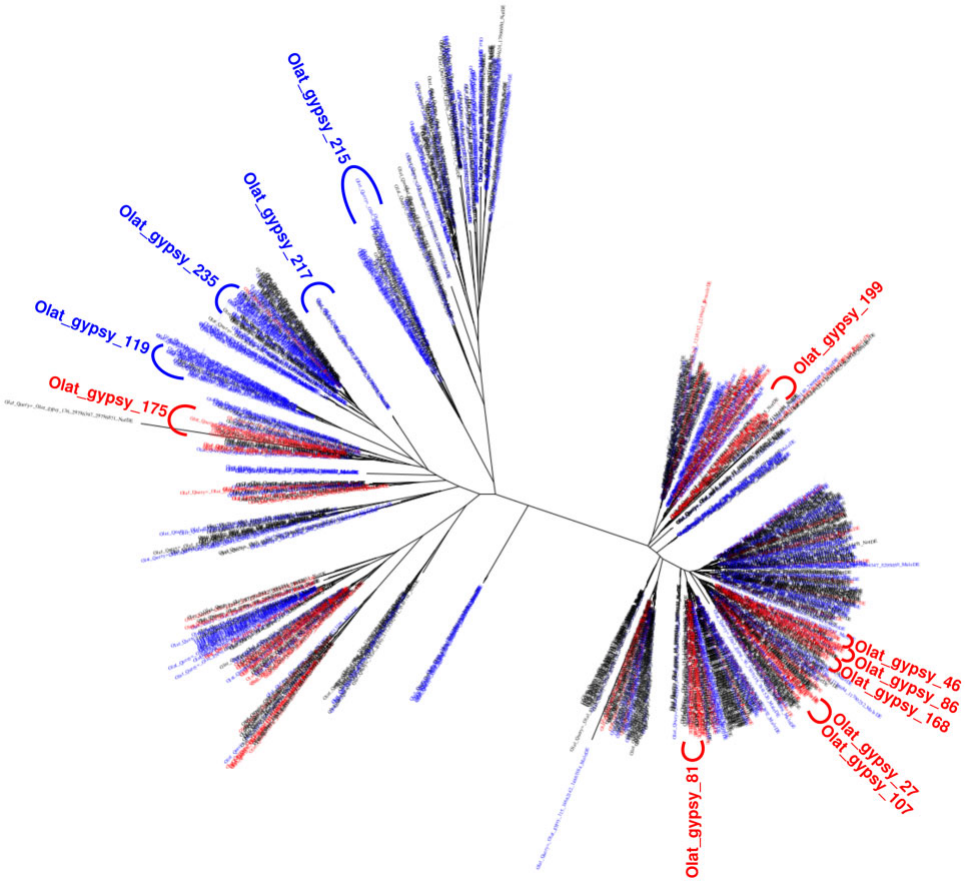
Phylogeny of expressed *Gypsy* TE copies of medaka using the amino acid sequence of the RT. Tip colors correspond to the expression bias (red: ovary; blue: testis; black: locus expressed but not biased). The location of biased *Gypsy* families is indicated. Note that copies with the same expression bias locally group together.

**Fig. 2.**
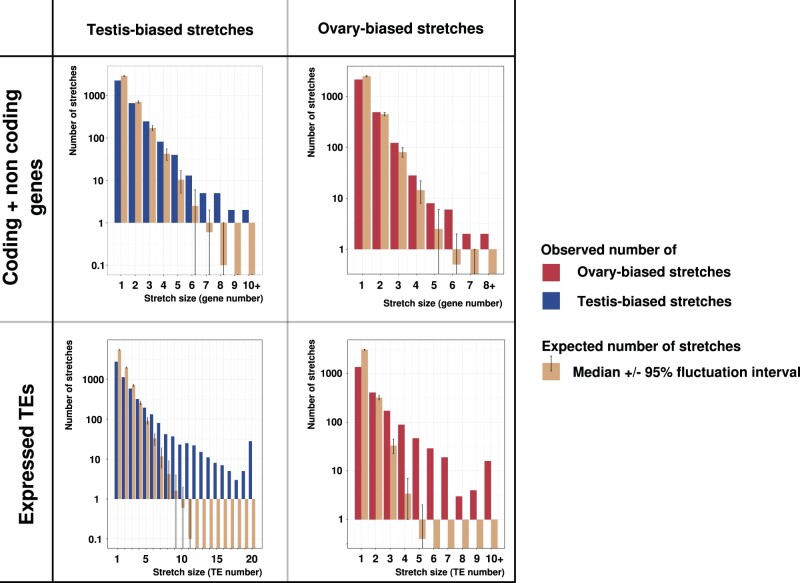
Stretches of consecutive sex-biased genes and TEs along the genome of *O. latipes*. First row: stretches of sex-biased genes. Second row: stretches of sex-biased TEs, as identified by SQuIRE. The first column represents testis-biased sequences, and the second ovary-biased sequences. The observed number of stretches of a given length (in gene number) in the genome are shown in red and blue for females and males, respectively. One thousand random genomes were generated using the same proportion of male- and female-biased genes or TEs to estimate the stretch sizes if genes or TEs were randomly distributed. The median number of expected stretches is shown in brown. Error bars represent the fluctuation interval containing 95% of the values generated by the bootstraps.

We further searched for TE families enriched in copies with testis- or ovary-biased expression. We found 22 families with global testis-biased expression (χ^2^ comparing the proportion of testis-biased copies in a given family to the proportion of testis-biased copies genome-wide: *P*<0.05/n_families and >50% of testis-biased copies), and 19 families with global ovary-biased expression (χ^2^ comparing the proportion of ovary-biased copies in a given family to the proportion of ovary-biased copies genome-wide: *P* value<0.05/n_families and >50% of ovary-biased copies, supplementary data 3 and fig. 2, Supplementary Material online) among a total of 1,164 families presenting at least one expressed copy. Interestingly, the majority of the sex-biased families correspond to class I long terminal repeats (LTR) elements: ovary-biased families comprise 13/14 LTRs and 1/14 long interspersed nuclear elements (LINEs), and testis-biased families 10/16 LTRs, 4/16 DNA transposons, 1/16 LINE, and 1/16 Rolling Circles. Only LTR elements are overrepresented among ovary-biased families compared with what would be expected from their genome-wide proportion (χ^2^, *P*<10^−4^). We generated a phylogeny of the LTR RT from family consensus sequences to confirm our annotation of the families, and to test if the expression pattern of the sequences is linked to their evolutionary relationships (supplementary fig. 3, Supplementary Material online). As most of the biased families are *Gypsy* elements, we more specifically generated a phylogeny of expressed *Gypsy* TE copies, irrespective of sex bias (fig. 1). We only used expressed TE copies for which we were able to detect an RT sequence. Plotting of expression patterns on the molecular phylogeny of TE copies showed that related TE copies often have similar sex-biased expression. To test if this could be explained by shared insertional preferences that would target similar TEs to similar expression environments, we analyzed sequences surrounding insertions of four subtrees (two mainly male-biased, one mainly female-biased, and one mainly non-biased; supplementary fig. 4, Supplementary Material online). The Guanine–Cytosine content was not significantly different between subtrees, and no insertion sequence specificity or preference could be detected (supplementary figs. 4 and 5, Supplementary Material online). These observations suggest that the expression bias may be explained by the sequence of the TE itself, that is that TEs would harbor regulatory sequences shared between phylogenetically related copies that co-regulate their expression. Analyzing *Gypsy* copies of a male-biased subtree of the previous phylogeny, we observed that at least 10 of them (42%) present LTRs and complete open reading frames (ORFs), suggesting that they are only mildly corrupted and might correspond to recent and autonomously transcribed insertions (supplementary fig. 5*A*, Supplementary Material online). However, the phylogeny also presents some examples of closely related copies with different expression patterns. This might be explained either by the loss of regulatory sequences by some copies, and/or by the influence of regulatory sequences from neighboring genes. We further drew a phylogeny including nonexpressed copies, but no particular clustering of expressed sequences compared with nonexpressed ones could be observed (supplementary fig. 5*B*, Supplementary Material online).

### Sex-Biased Genes and TEs Form Clusters in the Genome

To characterize the genomic distribution of sex-biased genes and TEs, we first asked if they physically clustered in the medaka genome. To unravel possible clusters of coexpressed genes, we applied a method that previously demonstrated the existence of large clusters of coexpressed genes in the Drosophila genome (Boutanaev et al. 2002). Briefly, stretches of adjacent genes or TEs with the same expression bias are counted across the genome. A stretch stops as soon as a gene or TE is found with a different expression bias: features within a stretch all present the same bias. For example, in the *O. latipes* genome, the biggest stretch of consecutive male-biased genes is 10 genes long (and spans 36 kb), and a maximum of 8 consecutive female-biased genes is found (spanning 133 kb). For TEs, a maximum of 32 and 15 male- and female-biased copies are found, respectively. The observed number of stretches was compared with an expected number computed from random distributions of genes in the genome (fig. 2, brown bars). We observed that testis- and ovary-biased genes are both not randomly distributed in the genome because they do not follow the expected random distribution in terms of stretches of adjacent genes with similar expression bias. We found more clusters of at least three genes than expected if genes were randomly distributed (fig. 2). Moreover, for testis-biased genes, we observed stretches of nine or ten genes, while such arrangement is never predicted among 1,000 random genomes. Results are given for coding and noncoding genes grouped together in the top panel of figure 2 (for coding and noncoding genes treated independently, see supplementary fig. 6, Supplementary Material online). We performed the same analysis with TE copies (fig. 2, bottom panel). We observed a nonrandom distribution for both testis and ovary sex-biased TEs as already found for genes. However, TE clusters contained markedly more elements than gene clusters: many comprised more than 8 TE copies, and up to 32 for male-biased TEs, while a maximum of 11 copies were predicted in a consecutive location in the random genomes. No particular enrichment of specific families could be observed in these clusters. As there are fewer ovary-biased TEs, the highest stretch sizes are lower than for male-biased TEs (15 and 32 consecutive biased TEs, respectively), but still higher than the maximum of 5 consecutive biased TEs expected at random under the null hypothesis. Globally, genes and TEs are thus not randomly distributed in the genome and tend to group into clusters with the same sex-biased expression. This is also true for coding and noncoding genes if analyzed separately (supplementary fig. 6, Supplementary Material online).

**Fig. 3.**
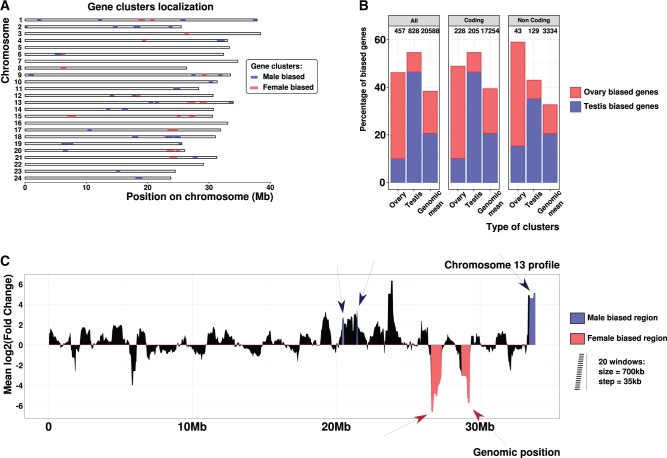
Sex-biased gene expression clusters in *O. latipes* genome. (*A*) Genomic location of gene clusters built from coding and noncoding genes. (*B*) Percentage of testis- or ovary-biased genes in each type of clusters. (*C*) Chromosome 13 profile obtained with *Gene clusters* representing the expression bias of all genes across sliding windows along the genome. The mean log_2_FC is represented for each window along the chromosome. The significantly male- and female-biased regions are shown in blue and red, respectively. The size of one window is shown, at scale, on the right of the plot.

As mentioned before, this first method of identifying clusters of coexpressed genes is quite inflexible, a single gene with a different expression profile disrupting stretches and potentially hindering the identification of interesting regions regrouping genes with the same expression bias. This is why we applied a second and complementary search to identify regions showing a high density of sex-biased genes or TEs. We used the *Gene clusters* method previously developed by our team (Toubiana et al. 2020), which calculates the local mean log_2_FC of the transcripts in a sliding window, and detects significantly biased regions (*P*_adj_< 0.05) using a bootstrap approach. We applied this to all gene transcripts, including coding and noncoding ones. We were able to identify 32 male-biased regions spread over 17 (out of 24) chromosomes (fig. 3*A*, supplementary table 1 and fig. 7, Supplementary Material online), and covering 3.94% of the genome. The method also uncovered 18 female-biased regions, spread over 13 chromosomes and covering 2.48% of the genome (supplementary table 1, Supplementary Material online, fig. 3*A*). Hence, about 6% of the genome of the medaka consists of sex-biased regions with respect to gene expression. In order to investigate their functions, we manually inspected all genes present in the sex-biased clusters in the medaka genome. We recovered genes known to be involved in male sexual functions in male-biased clusters, including *dmrt1a* (the autosomal paralog of the master sex-determining gene *dmrt1bY*), *morn3*, *frizzled-4*, *ucp2*, and *lrguk*, and genes with known female sexual functions in female-biased clusters, such as *zonadhesin*, *bucky ball*, *bokb*, and *hsd17b1* (supplementary table 2, Supplementary Material online). We also tested the association of genes in sex-biased clusters with Gene Ontology (GO) terms. At the genome-wide scale, male-biased genes were significantly associated with “spermatogenesis,” “cilium assembly and function,” and “protein polyglutamylation” (Bobinnec et al. 1999, supplementary fig. 8, Supplementary Material online), and female-biased genes with “acrosome reaction,” “oogenesis,” and “binding of sperm to zona pellucida” (supplementary fig. 9, Supplementary Material online), indicating a global link in our data between sex-biased expression and gonadal/germ cell function. In contrast, only the term “spermatogenesis” was significantly associated with genes in male-biased clusters (supplementary fig. 10, Supplementary Material online), and no significant GO term linked to sexual function and reproduction was found for genes in female-biased clusters (supplementary fig. 11, Supplementary Material online). In addition to the low power in the GO enrichment analysis due to a limited number of genes, this might also indicate that genes in sex-biased clusters, compared with sex-biased genes in general, are enriched in genes with so far uncharacterized sexual functions (possibly lineage-specific and evolving more rapidly), with functions that are less sex-specific than germ cell functions, or with functions that have been more recently recruited to the gonads.

We called “gene clusters” the regions presenting a significantly higher mean differential expression of genes. Eight hundred ten sex-biased genes (3.9% of all genes, and 10.1% of all sex-biased genes) are located in a cluster with the same expression bias. The size and gene composition of these regions calculated using coding genes only, noncoding genes only, or both are described in supplementary table 1, Supplementary Material online. Among the 828 genes located in a testis-biased region, 353 (42.6%) are testis-biased, whereas 78 (9.4%) are ovary-biased (fig. 3*B*). Ovary-biased regions contain 457 genes with 165 (36.1%) female-biased genes and 47 (10.3%) male-biased genes (fig. 3*B*). As an example, we observe on chromosome 13 of *O. latipes* two main ovary-biased regions of ∼1 Mb with a mean log_2_FC of –5 (fig. 3*C*). For the detailed gene cluster profiles for each chromosome see supplementary data 4–7, Supplementary Material online.

In contrast to the situation observed in Drosophila (Boutanaev et al. 2002; Ellison and Bachtrog 2013), we did not observe any particular trend on chromosome 1, which is the X chromosome. The Y chromosome of *O. latipes* differs from the X by a 250-kb insertion that includes only one gene, the master sex-determining gene *dmrt1by*. The rest of the X and Y chromosomes are very similar. The Y-specific region is not represented in the reference genome we used (thus containing an X but not a Y chromosome; https://www.ncbi.nlm.nih.gov/assembly/GCF_002234675.1, last accessed September 2021). If we look at the cluster profile obtained from TE expression, we observe on the X chromosome a testis-biased TE cluster surrounding the insertion breakpoint of the Y-specific region (supplementary data 7, chromosome 1, and fig. 12, Supplementary Material online). This cluster was thus present next to the region of insertion, and the insertion seems to have occurred in a region that was already male-biased.

### Neighboring Genes and TEs Share Correlated Expression Bias between Male and Female Gonads

**Fig. 4.**
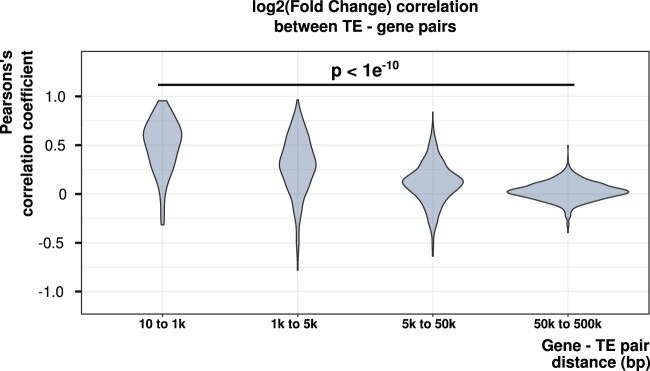
Correlation coefficients calculated on expression bias between adjacent genes and TE copies for different intervening distances, for each TE family. Each violin corresponds to the distribution of correlation coefficients of the different TE families. Fewer gene–TE pairs are found for the shortest distance categories, explaining why the variance is higher for these categories. The closer TEs and genes are located, the higher the correlation coefficient of expression bias between genes and TEs.

As we could determine the expression of TEs at the copy level, we next asked if neighboring genes and TEs shared similar expression patterns that would reflect a coregulation (originating from an enhancer of the gene or from the TE, or from the epigenetic state of the whole region). To do so, we assessed whether the sex bias in expression of adjacent genes (coding and noncoding: fig. 4, coding or noncoding: supplementary fig. 13, Supplementary Material online) and TEs is correlated. Only genes and TEs expressed in gonads were used to test the hypothesis. We calculated the Pearson correlation coefficient of log_2_FC of gene–TE pairs with different distance categories (border to border gene–TE distance 10 pb–1 kb, 1–5 kb, 5–50 kb, and 50–500 kb). A given TE copy can be associated to all genes within the distance selected, and conversely a given gene can be associated to all TEs in the distance selected. We calculated the correlation using the copies grouped by TE family for families containing at least five TE copies, and making at least ten gene–TE pairs (i.e., ten TEs with one gene, or five TEs with two genes). The correlation coefficient of each family is represented depending on the distance considered to create the gene–TE pairs (fig. 4). We observed that the closer TEs and genes are located, the higher the correlation coefficient is (one-way analysis of variance, *P*<1e–10). The same result was obtained using only the coding or the noncoding genes (supplementary fig. 13, Supplementary Material online).

We complementarily asked if clusters of sex-biased genes could concentrate TEs with the same sex bias in gonadal expression. No clear common pattern of gene and TE distribution emerged from the inspection of gene clusters, indicating the diversity of their structures. In some clusters hotspots of TEs were observed (e.g., supplementary fig. 7*A*, Supplementary Material online), whereas in others, TEs showed no marked local enrichment (e.g., supplementary fig. 7*B*, Supplementary Material online). On average, TE copies located in testis-biased regions are overexpressed in testis (mean log_2_FC=2.96), and significantly more than TEs not located in sex-biased clusters (mean log_2_FC=1.35, Student’s *t*-test *P*<1e–16). TEs located in female-biased regions also present on average overexpression in testis (mean log_2_FC=0.77), but at a lower level than TEs not located in sex-biased clusters (mean log_2_FC=1.35, Student’s *t*-test *P*=4.2e–6; supplementary fig. 14, Supplementary Material online). As the number of copies compared between both groups is large, a low *P* value is not really informative, and it is important to check for the size of the effect (mean log_2_FC 0.77 vs. 2.95 vs. 1.35). TEs located in ovary-biased regions are still more testis-biased than ovary-biased, which reflects the previous observation that the general trend of the genome is a testis-biased expression of TEs. Of the genome, 3.94% and 2.48% are considered as male and female sex-biased gene clusters, respectively; 5.84% of expressed TEs and 4.11% of all TEs (expressed or not) are located in male-biased gene clusters, whereas 1.81% of expressed TEs and 2.40% of all TEs are located in female-biased gene clusters. Overall, the results indicate that globally TEs do not preferentially integrate into sex-biased clusters (this was also observed considering only recent insertions, to take into account the possibility that clusters change their position during time; data not shown). A slight enrichment might be observed for expressed TEs in male-biased clusters, which might reflect preferential insertion (maybe due to open chromatin in testis) and/or positive selection of insertions in these regions, but this minor effect requires further investigation to assess its significance. To double-check for the presence of sex-biased TEs in sex-biased regions, we investigated if sex-biased TE copies were more likely to be inserted in regions with a similar sex-bias gene expression than somewhere else in the genome. For that, we tested the possible relationship between the localization of the copy and its expression (supplementary fig. 15, Supplementary Material online). Most of the 37,038 TE copies analyzed are located in unbiased regions and present unbiased expression (supplementary fig. 15, Supplementary Material online, 18,884 copies). We computed the expected copy numbers if there were no association between both localization and expression (supplementary fig. 15, Supplementary Material online, gray values), and tested the difference between observed and expected counts using a χ^2^ test of independence (*P*<1e–125). Again, as the number of copies is very high, it is important to consider the size of the effect (supplementary fig. 15, Supplementary Material online, ratio values) and not only the *P* value. We observed an enrichment of 1.72 (1,293/773) for testis-biased TE copies present in testis-biased regions, showing that there are more testis-biased TEs in testis-biased regions than expected at random. For ovary-biased copies within ovary-biased regions the enrichment is 1.58, which means that there are more ovary-biased TEs located in ovary-biased gene clusters than expected at random. On the contrary, testis-biased regions should contain approximately 217 ovary-biased copies, but contain only 114, making these regions depleted in ovary-biased copies (fold change ∼0.53). Conversely, only 163 testis-biased copies are found in ovary-biased gene clusters (vs. 215 expected). Thus, these regions are depleted for testis-biased TEs (fold change ∼0.76).

**Fig. 5.**
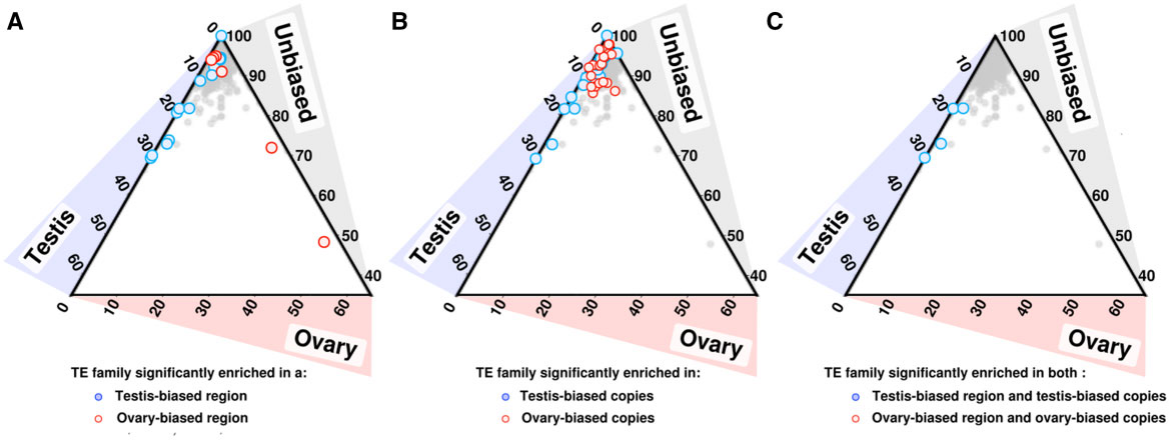
TE copies from specifically testis-biased TE families are preferentially located in testis-biased gene clusters. (*A*) Comparison of location and expression of TE families. Each data point corresponds to one TE family. The gray scale gives the percentage of copies of the family located in an unbiased genomic region; on the left the percentage of copies of the family located in a testis-biased genomic region; and on the bottom, the percentage of copies of the family located in an ovary-biased genomic region. TE families significantly enriched in the biased regions are shown in red or blue. (*B*) The same data as in *A* but with a different coloration: TE families are highlighted in red and blue if their copies are significantly more expressed in one sex compared with the other. Families enriched in male-biased regions are often enriched in male-biased copies. In contrast, families enriched in female-biased regions are not necessarily made up of copies overexpressed in ovaries. (*C*) Intersection between *A* and *B*. The five TE families with both significant testis-biased expression and testis cluster localization are shown in blue.

Finally, we tested if TE families harboring a large proportion of sex-biased copies are more likely to be located in sex-biased regions. Independently of the expression bias of their copies, and taking into account both expressed and nonexpressed copies, 19 TE families were enriched in testis-biased regions, whereas 9 families appeared enriched in ovary-biased regions (fig. 5*A*). In figure 5*B*, we show the same data but coloring TE families if the expression of their copies is significantly biased in one sex. Overlapping the two graphic representations of the data (fig. 5*C*), we observe that four of the families enriched in male-biased regions correspond to families for which copies present a strong biased expression toward males (supplementary data 3, Supplementary Material online). These families include a family of *Helitrons*, which are class II TEs involved in dosage compensation in Drosophila males (Ellison and Bachtrog 2013, 2015), and are preferentially localized in the sex-determining region of the platyfish *Xiphophorus maculatus* (Zhou et al. 2006). Another of these four families is an Unknown TE family that is highly testis-biased, with 51 of 56 expressed copies being more expressed in testis than in ovary, the remaining 5 being nonbiased. This family is enriched in testis-biased regions, with 16% of the copies located in such regions (15/93 copies in total, and 8/56 expressed copies). We analyzed the sequence of the copies from this family. We found two putative transcription factor binding sites (TFBS) enriched in expressed copies compared with nonexpressed ones (supplementary fig. 16, Supplementary Material online). These transcription factors are involved in male gonad development, Sertoli cell development, and spermatogenesis (SOX8), and in response to testosterone and male genitalia development (HOXD13). Among the six TE families preferentially located in female-biased regions (fig. 5*a*), there is no family significantly harboring female-expressed copies (fig. 5*c*).

## Discussion

### Global Gene Expression Analysis Reveals a Similar Proportion of Testis- and Ovary-Biased Coding Genes in the Medaka

In this study we investigated gene and TE expression in the gonads of the Japanese medaka *O. latipes*. Compared with other tissues, gonads are the most sex-biased organs in terms of gene expression (Böhne et al. 2014; Tsakogiannis et al. 2018). These organs thus constitute a good model to study sex-dependent gene expression regulations. As the dissection of medaka gonads does not allow physical separation of the germline from the soma, our data comprise both types of cells and should be interpreted as a gonadal and not as a germline expression analysis. Several studies previously analyzed RNA-seq data in teleost fish, mainly from gonads (Böhne et al. 2014; Liu et al. 2015; Robledo et al. 2015; Bar et al. 2016; Zeng et al. 2016; Wang et al. 2017; Tao et al. 2018; Tsakogiannis et al. 2018) or from brain (Böhne et al. 2014; Liu et al. 2015; Saaristo et al. 2017; Beal et al. 2018; Tsakogiannis et al. 2018; Wu et al. 2019; Shen et al. 2020). Here we found that 40% of protein-coding genes present a sex-biased expression in medaka gonads, with 21% being testis- and 19% ovary-biased. In cichlid fish, by analyzing four species, 66% of the coding genes were found differentially expressed, with slightly more testis-biased genes (Böhne et al. 2014). In the Japanese fugu, only 3.7% of the coding genes were found differentially expressed between gonads, but still with more testis-biased genes (Wang et al. 2017). The proportion of gonad-biased genes thus greatly varies between fish species and probably also depends on the conditions and parameters of analysis. Many technical biases could explain these variations, because RNA-seq data analysis relies on several parameters including the type of pipeline used, the availability of a reference genome of good quality, and the thresholds applied to decide if a gene expression is sex-biased. The observed variations could nevertheless also result from true biological variability, notably because of differences in gonad maturity between species: medaka is a constitutive spawner, whereas fugu and cichlids have breeding periods, and the bluehead wrass is a consecutive hermaphrodite. A common trend that persists is the slight bias toward testis overexpressed genes. However, this effect is not that important in medaka, and we can consider that genes are overexpressed in similar proportion between sexes in this species.

### A Higher Proportion of TEs and Noncoding RNA Genes Are Overexpressed in Testis Compared with Ovary

As TEs are known to be particularly involved in the regulation of vertebrate noncoding RNAs (Kapusta et al. 2013), we included noncoding RNA genes in our analyses, either merged with protein-coding genes, or as a distinct class of genes. It should be noted that, because our sets of transcripts were generated using poly-A purification and because a large part of noncoding RNAs is not polyadenylated (Cheng 2005; Kapranov et al. 2010; Sun et al. 2018), noncoding genes are likely to be underrepresented in our data set. Although RNA genes were globally found to be slightly less differentially expressed than coding genes (33.2% vs. 40%, respectively), they appeared more biased toward testis (63% of differentially expressed noncoding RNA genes are testis-biased, vs. 52% of differentially expressed protein-coding genes). Some of them might correspond to lncRNAs important for spermatogenesis, as reported in tetrapods (Necsulea et al. 2014). PolyA-enriched RNA-seq data are also relevant to study TE expression (Lerat et al. 2017; Lanciano and Cristofari 2020), as both class 1 and class 2 TEs express polyadenylated RNAs (Deininger and Batzer 2002). Almost half of expressed TE insertions were found differentially expressed (46.3%), with a major bias toward testis compared with ovary (78% vs. 22% of biased TEs, respectively). It is expected that TEs are particularly expressed in the germline, because their spreading and fixation in the genome rely on their activity in these cells (Dechaud et al. 2019). The question may arise if the higher proportion of testis- versus ovary-biased TEs might be a consequence of the different proportions of germline and somatic cells in the male and female gonads. These proportions are not available so far from literature, and we can only roughly estimate from our own unpublished observations in medaka a ratio of soma versus germline cells of ca. 1:100 in testis and 100:1 in ovary. Male and female germ cells, however, do not contain the same amounts of RNA. Again, there are no data available concerning this point, but we can very roughly estimate the ratio of soma versus germ cell RNA amount to be of 1:100 in both types of gonads. This estimate is reinforced by the similar proportions of male- versus female-biased gene transcripts determined in gonads, suggesting that expression levels are not much biased by their intrinsic structure. The higher proportion of testis-biased TEs could rather suggest that in medaka the male gonad is more permissive than the female gonad in terms of TE expression. This idea is somehow in opposition to the observations concerning the control of TEs in fish by piRNAs, a class of noncoding RNAs molecules that induce TE mRNA degradation. Indeed, both in zebrafish (Houwing et al. 2007) and medaka (Kneitz et al. 2016), piRNAs are more expressed in testes than in ovaries. Consequently, we might expect lower levels of TE expression in testis compared with ovary. However, the steady-state TE mRNA levels we observe result from both transcription and repression. The higher TE expression measured in testis could therefore result from much higher initial transcription, somehow counterbalanced by piRNA-mediated repression. More precise investigations are needed to conclude on this point. In addition, some retrotransposon families were shown to escape methylation in testis in mammals (Molaro et al. 2011). Similar experiments are needed in fish to evaluate if differential methylation of TEs might be linked to their higher level of expression in testis compared with ovary.

### Sex-Biased Genes and TEs Form Clusters in the Medaka Genome

We focused particularly on the relative localization of genes and TEs with respect to their sex-linked expression bias. Many studies already reported that sexual genes (Boutanaev et al. 2002; Li et al. 2005; Dorus et al. 2006) and genes in general (Lercher et al. 2002; Roy et al. 2002; Singer et al. 2005) are not randomly distributed on chromosomes. Regulatory constraints such as the presence of specific enhancers or local chromatin states that affect neighboring genes can, for instance, result in the conservation of syntenies, even between distant species (Ferrier and Holland 2001; Kikuta et al. 2007; Pascual-Anaya et al. 2013). These functional constraints are now known to translate into topologically associated domains (Jordan Rowley and Corces 2018; de Wit 2019), which are regions preferentially interacting in the 3D organization of the genome. A way to define clusters is by counting successive genes presenting the expression pattern (or any other feature) of interest (Boutanaev et al. 2002; Lercher et al. 2002; Roy et al. 2002; Li et al. 2005; Singer et al. 2005; Dorus et al. 2006). Such a procedure is quite stringent, but can reveal candidate regions where large stretches of coregulated genes exist. Applying this approach, we showed that the *O. latipes* genome is enriched in stretches of more than three sex-biased genes, this observation being valid for both coding and noncoding genes. When considering TEs, the enrichment is even stronger, with stretches of more than 20 testis-biased elements. This suggests that some regions contain a higher density of sequences with sex-biased expression.

In a second and complementary attempt, we developed a new method that evaluates the global expression bias of genes along the chromosomes and identifies regions with a mean expression bias significantly diverging from random expectations. This method can be applied to any set of differentially expressed genes, and not only to sex-biased genes, the only requirements being the localization of the genes and the associated log_2_FC of their transcripts. Applying this sliding-window approach, we detected 32 and 18 clusters enriched in genes with male- or female-biased expression, respectively. These clusters contain about 10% of all sex-biased genes. We can hypothesize that such organization is linked to a common regulation of the clustered genes. The notion of “synexpression groups” has been proposed in eukaryotes, which corresponds to sets of coregulated genes (Niehrs and Pollet 1999; Ramialison et al. 2012; Herpin et al. 2019). In the medaka, a pilot study including 560 genes expressed during embryonic development showed that coregulated genes tend to share particular DNA motifs in their *cis*-regulatory regions (Ramialison et al. 2012). These motifs allow the genes to be tightly controlled in space and time during development. About a third of the developmental syn-expression groups presented pairs of genes distant from less than ten genes, which is rarely observed by chance (Ramialison et al. 2012). This revealed a slight tendency of coregulated genes to group on the chromosomes. With the finding of 50 sex-biased gene clusters encompassing 25 biased genes in the mean, our genome-wide analysis made a big step in this observation. It also demonstrates that this trend not only concerns developmental genes but also genes functioning in mature organs. It would be interesting to compare the domains we identified with data of Hi-C, for now unavailable for medaka gonads (Nakamura et al. 2021).

### A Cluster of Sex-Biased TEs Could Have Favored the Birth of Sexual Chromosomes

In mammals, X and Y chromosomes stopped recombining 210 Ma (Waters et al. 2007), leading to an accumulation of TEs and a loss of genes on the Y chromosome. In *O. latipes*, the sex chromosomes are relatively young. X and Y chromosome are still recombining on their almost complete length and their single main structural difference is a short Y chromosome-specific region of 250 kb containing the master sex-determining gene *dmrt1by* and its transcriptional regulatory region, a copy of the *Izanagi* transposon (Kondo 2006). One theory predicts that an important early step in the evolution of the Y chromosome is the linkage of sexually antagonistic genes that are beneficial to males but not to females in the Y-specific region (Charlesworth et al. 2005). This leads to a loss of recombination between the X and Y chromosomes in this region, and in consequence to the accumulation of TEs that can no longer be purged by crossing-over with the homologous region lacking the TEs (Charlesworth et al. 2005). We found on the X chromosome a testis-biased cluster of TEs surrounding the insertion breakpoint of the Y-specific region, suggesting that the future Y chromosome of *O. latipes* already accumulated male-biased TEs in this region before the new master sex-determining gene was inserted. It is thus tempting to speculate that this region enriched in sex-biased TEs could have eased the recruitment and evolution of the new male master-sex determining gene *dmrt1by* of *O. latipes* by providing a favorable male transcriptional environment.

### Disentangling the Possible Functional Links between TEs and Sexual Genes

It has been already established that TEs are generally not randomly distributed in the genome. Some retrotransposons, for instance, are able to target regions (either precise nucleotide sequences or larger particular chromatin environments) where they can insert without generating deleterious mutations, thus limiting their counterselection (Sultana et al. 2017). Some *Ty* retrotransposons target the upstream region of pol-III transcribed genes in *Saccharomycescerevisiae*, which allows both their expression and their location in a “safe” environment, with no risk of disrupting essential genes (Guo et al. 2015; Spaller et al. 2016; Cheung et al. 2018). Spreading and fixation of TEs is intrinsically linked with their activity in gonads, and more precisely in germ cells (Dechaud et al. 2019). They could be positively selected if they insert in a region allowing their expression in this tissue. Postintegration selection is also an important force modulating the location of TEs. Strongly deleterious insertions, such as insertions disrupting essential genes, are rapidly removed from the genome because individuals carrying them are strongly disadvantaged (Medstrand et al. 2002). In contrast, insertions with a positive impact on host fitness will be retained by selection. TEs are now known to be able to modulate gene expression and to rewire entire regulatory networks (Feschotte 2008; Chuong 2013). It has already been proposed that TEs harboring TFBS allowing their germline expression could serve as a “taxi” for regulatory elements to also control the expression of surrounding genes (Rebollo et al. 2012; Sundaram et al. 2014; Dechaud et al. 2019). They thus constitute good candidates to be involved in the fast evolution of sexual pathways in medaka. If such a positive selection concerns several insertions of the same TE family, these insertions can concomitantly appear enriched in different regions where they bring advantages. Finally, most insertions have limited impact on host fitness; they generally undergo genetic drift and are eliminated through random mutations. The combination of all these mechanisms can ultimately lead to an enrichment of TEs in particular genomic regions, either due to insertional preferences, to their low impact in these regions, or on the contrary to an acquired positive functional role, for instance in host gene regulation. To get more insights into these different evolutionary processes, we asked if the location of sex-biased TEs in *O. latipes* could be related to that of sex-biased genes.

We showed here that the sex-biased expression of neighboring genes and TEs is correlated: the closer TE copies are to genes, the higher is the correlation of their expression. Interestingly, such a coexpression has been also observed for TEs located near antiviral response genes in human and mice cells, suggesting that this phenomenon might not be restricted to sexual genes (Macchietto et al. 2020). This observation allows hypothesizing a coregulation of both types of sequences by shared *cis*-regulatory elements, provided either by enhancers of the sexual genes and/or by the sex-biased TEs themselves. Both hypotheses are not mutually exclusive and further analyses will be necessary to understand the origin of this correlation. In the first hypothesis, TEs inserting close to sexual genes could co-opt regulatory sequences favoring their expression and by this way their transposition in gonads, particularly in germ cells for transmission to the next generation. Our observation that expressed TEs are slightly enriched in male-biased clusters could indeed reflect a preference of insertion of TEs in these regions. The effect, however, was minor, and not observed for female-biased clusters.

In our data, TE families enriched in copies mainly expressed in one sex are LTRs, for both testis and ovary. Endogenous retroviruses (ERVs) were previously shown to frequently give birth to enhancers in fast-evolving tissues (Simonti et al. 2017), and more particularly their LTRs that contain many TFBS (Teng et al. 2014; Thompson et al. 2016). Furthermore, LTR elements are known to escape repression in tissues such as testis or placenta, which are hypomethylated and thus allow a higher global transcriptional activity (Molaro et al. 2011; Chuong 2013). However, further experiments would be needed to demonstrate such a recruitment of LTR elements for regulatory purpose. Even if we could not identify any sequence insertion preference between related *Gypsy* elements with the same sex-biased expression, from our data we cannot completely eliminate a purely neutral model where TEs preferentially insert in regions of open chromatin and subsequently follow the expression of neighboring genes. About three times more TEs were found overexpressed in testes than in ovaries. This observation agrees with the transcription of various genomic elements known to occur in testis (Soumillon et al. 2013). If the majority of these transcripts are probably nonfunctional, this high level of expression could also favor the birth of new genes or regulatory elements in this organ, in particular from TEs (Simonti et al. 2017).

Finally, we demonstrated in our work that sex-biased TE copies are enriched in gene clusters with the same sex-biased expression. We particularly identified an Unknown TE family strongly biased toward testis expression, and preferentially localized in regions considered as male-biased. Of note, expressed copies of this family are enriched in binding motifs for SOX8 and HOXD13, two factors involved in male sexual function. This TE family constitutes thus an interesting candidate to investigate for a potential role in the evolution of sex chromosomes and/or the regulation of sexual development of the medaka fish.

## Materials and Methods

### Experimental Animals

Laboratory-reared medaka (*Oryzias latipes*) of the Carbio strain were used. All fish were kept under standard photoperiod cycle of 14 h/10 h light/dark at 26 °C (±1 °C). Animals were kept and sampled in accordance with the applicable EU and national German legislation governing animal experimentation, in particular all experimental protocols were approved through an authorization (568/300–1,870/13) of the Veterinary Office of the District Government of Lower Franconia, Germany, in accordance with the German Animal Protection Law (TierSchG).

### Sampling and Sequencing

The gonads of *O. latipes* were dissected. As testes are small, the testes of three males were pooled in one replicate. We generated three testis replicates (3 × 3 fish) and three ovary replicates (3 × 1 fish). Total RNA was isolated using RNeasy Mini kit (Qiagen) following the manufacturer’s instructions. RNA quality was assessed by measuring the RNA Integrity Number (RIN) using an Agilent 2100 Electrophoresis Bioanalyzer Instrument (Agilent Technologies 2100 Expert). RNA samples with RIN>8 were used for sequencing. RNA sequencing libraries were constructed following the standard TruSeq Illumina mRNA library preparation protocol (www.illumina.com, last accessed November 2019; Illumina Inc., BGI, Hong Kong), with a read length of 100 and sequencing depth for paired end of 62–72 million reads.

### Genome and TEs Annotation

The genome of *Oryzias latipes* strain Hd-rR was sequenced and assembled with chromosome length scaffolds (https://www.ncbi.nlm.nih.gov/assembly/GCF_002234675.1, last accessed September 2021). This genome is also annotated (https://ftp.ncbi.nlm.nih.gov/genomes/refseq/vertebrate_other/Oryzias_latipes/latest_assembly_versions/GCF_002234675.1_ASM223467v1, last accessed September 2021).

TEs were annotated using the following protocol. First, a TE consensus database was generated using RepeatModeler (http://www.repeatmasker.org/RepeatModeler/, last accessed May 2021; 1,596 consensi). To avoid false positives we removed short consensi under 80 nt (1,400 resulting consensi), we self-blasted each consensus to find potential satellite sequences (1,398 resulting consensi), we removed non-TE genes by blasting the consensi against NCBI (13,82 resulting consensi), and we removed the redundant consensi by blasting the bank against itself (947 consensi). We crossed the bank with LTRharvest (Ellinghaus et al. 2008) output to reannotate some ERV TEs, and added *Gypsy*, ERV, and *Copia* elements (1,262 resulting consensi). We also added two *Helitron* sequences from HelitronScanner (Xiong et al. 2014) that were not found by other programs (1,264 consensi). Some SINE sequences were reannotated using SINE_scan (Mao and Wang 2017). We finally ran MITE-hunter (Han and Wessler 2010), but after manual checking we did not find any good consensus to add. The bank was used to annotate the genome with RepeatMasker (http://repeatmasker.org/, last accessed May 2021). All TE copies annotated by the same consensus sequence are considered part of the same TE family.

### Gene Expression Analysis

A detailed description of our protocol along with all parameters applied here is listed in supplementary file supp_data.odt, Supplementary Material online. Read mapping was performed with Hisat2 version 2.1.0 (Kim et al. 2019). As, at this step, we wanted to exclude from the assembly potentially expressed TEs, we discarded multimapped reads. We then used *StringTie* (Pertea et al. 2015) to assemble the transcripts using the genomic coordinates of the aligned reads, and to quantify transcript expression in each sample. We used the *ballgown* R package (Frazee et al. 2015) to estimate the transcript per million expression of each gene or transcript. Transcripts with low expression were filtered out as recommended in the *new tuxedo* procedure (Pertea et al. 2016). Genes and transcripts reconstructed by *StringTie* (Pertea et al. 2015) were compared with the reference gene annotation of the genome. Each reconstructed transcript was assigned a class code depending on its similarity to a reference transcript, allowing identification of whether it was already present in the reference or if it is new.

### TE Expression Quantification

We used SQuIRE (Yang et al. 2019) (https://github.com/wyang17/SQuIRE, last accessed September 2021) to estimate TE expression at copy level resolution. This program does not count reads multimapped several times that could be assigned to several (highly similar) TE copies. Using different parameters such as local proportions of uniquely mapped reads, at the end attributes each read to a specific locus, or “shared” it between different loci but with a divided score. SQuIRE thus does not overestimate the expression of young TE families containing several highly similar copies. SQuIRE is divided into different steps to perform its analysis. The “Fetch” step retrieves the genome of interest along with gene and TE annotations on the University of California–Santa Cruz (UCSC). As the *O. latipes* genome available on UCSC is not up to date, and as we build our own TE library, we did not use this step and generated the corresponding files using the latest *O. latipes* genome and our TE library. We then ran SQuIRE “clean,” “map,” “count,” and “call” steps to estimate TE expression.

### RT Phylogeny

We first defined a reference set of RT amino-acid sequences from a subset of the different LTR consensus sequences identified in the medaka genome for the different LTR superfamilies (*Gypsy*, *Copia*, ERV, and *BEL*/*Pao*). Using ORFfinder (https://www.ncbi.nlm.nih.gov/orffinder/, last accessed May 2021) and conserved domain detection (https://www.ncbi.nlm.nih.gov/Structure/cdd/wrpsb.cgi, last accessed May 2021), we obtained the amino-acid sequence of their RT. We stored these sequences in *Reference_RT.fa* (supplementary data 8, Supplementary Material online). We then compared all LTR elements with these reference RTs by blastx. Using *recup_prot_query_Blastx.py* we retrieved for each TE the best hit of more than 50 amino acids. We added some known RT from other species found on NCBI: *RT_ref.fa* (supplementary data 9, Supplementary Material online). These sequences were aligned with *mafft* (version v7.450; https://mafft.cbrc.jp/alignment/software, last accessed January 2020; Katoh 2002; Katoh and Standley 2013), and stored in *RT_ref.mafft* (supplementary data 10, Supplementary Material online). This alignment was fed to *FastTree* (version 2.1.11; Price et al. 2010) with Le and Gascuel (LG) model to build the shown phylogeny (supplementary data 11, Supplementary Material online). The different codes for blastx or *mafft* used to generate the phylogeny are described in *Phylogeny.md* on the gitlab repository (see below). We used the exact same approach to design the tree using expressed *Gypsy* copies (supplementary data 12 and 13, Supplementary Material online).

### Building Gene and TE Stretches

The method used to generate figure 4 is the same as described in Boutanaev et al. (2002). The genes of TEs are represented by their expression bias, either testis, “T,” ovary, “O,” or nonbiased, “N.” They are represented as a sequence on each chromosome, like, as an example:

–NNN**OO** NN**T**NN**TTT**NNN**TO**NN**TOT**NNN**TTTTT**N**O**NN**O**NN**T**N–

For each bias, the number of stretches is then counted. In this example, for testis-biased genes, we observe five stretches of one gene, one stretch of three genes, and one stretch of five genes. This will be represented by the blue bars in figure 4. Then the genome is shuffled 1,000 times. For each shuffling, the same counts are computed and we finally plot the median value with the 95% fluctuation interval. This is not a confidence interval of the mean, but represents the distribution of 95% of the values obtained through the shuffles. The same approach was applied for ovary-biased genes. The script used to generate such a barplot is available from the gitlab repos (see below: *stretch_of_genes.R*).

### Detection of Gene Clusters

The search for such stretches, however, is not fully suitable to detect genomic regions enriched in biased genes. If two consecutive genes are separated by a long gene desert, for example, they can still belong to a common stretch, in spite of their important intervening distance. Additionally, a single gene with a different expression can split a cluster, hindering its identification in spite of the global common expression bias of surrounding genes. It is thus interesting to study gene clustering on chromosomes in a more relaxed manner. We thus developed a new method to determine if genes are randomly distributed on chromosomes, or if they are grouped according to their expression, by designing a bootstrapping approach. The pipeline is available at https://gitlab.com/Corend/gene_clusters_pyth (last accessed October 2021), and was already used in a study on waterstriders (Toubiana et al. 2020).

#### Step 1: Design of the Expression Profile

First, a sliding window is designed on the genome. The size and step of the sliding window can be set by the user through the *-step* and *-window* parameters in the pipeline. The window and step sizes have to be chosen carefully according to the gene density of the studied genome, to ensure a sufficient statistical power. We tested different values and retained a step size of 35 kb and a window size of 700 kb. Hence, each window overlaps with the next 20 windows. Then, using the bed file of genes provided by the user (*-b*) and the expression of each gene (*-e*), the mean log_2_FC of the genes is calculated in each window of each chromosome. The result can be used to display the fold change across each chromosome (fig. 3*C*).

#### Step 2: Bootstrap Analysis

Randomly distributed genes can form clusters just by chance. To test whether a cluster is observable by chance, we designed a bootstrap approach. The bootstrap number can be adjusted by the user (*-boot*). We used 10,000 bootstraps for our analysis. For each bootstrap, all the genes in the genome are randomly redistributed at each locus. Then the mean log_2_FC is calculated again in each window. After the bootstraps, we have for each window the observed mean log_2_FC, and 10,000 theoretical mean log_2_FC. We calculate how many times the observed value is superior to the bootstrap and how many times it is inferior. If the observed value is always superior to the bootstrap values, then this region can be considered as “significantly testis-biased.” On the other hand, if the observed value is inferior to the bootstraps, it is considered as “ovary-biased.” The output of the pipeline corresponds to the mean log_2_FC for each window and its associated bootstrap value.

#### Step 3: Statistical Analysis

This part is not included in the pipeline so that the user can choose its own bootstrap threshold. We converted the bootstrap value in a *P* value: [min(Bootstrap superior, Bootstrap inferior) × 2]/10,000. We then converted the *P* values (1 *P* value per window) in *q* values using Benjamini–Hochberg false discovery rate correction from the R package *qvalue* (Storey et al. 2019). We use a *q* value threshold of 5%, meaning that among the windows considered as enriched in biased genes, 5% are false positives. Using this threshold, we colored the regions on the plot (fig. 3*C*).

### Enrichment of TEs in Biased Regions

We investigated the localization of the TE copies of each family (fig. 5). We tested if each family was significantly enriched in the ovary- or testis-biased regions using Fisher’s exact test according to the method described in Karakülah and Suner (2017). We tested the association between TE copy location and TE family expression using a Fisher exact test. As we tested all the TE families (1,164), we applied a Bonferroni correction to the test by taking 0.05/1164 as a *P* value threshold. The ternary plot was generated using the script *Ternary_plot.R.* The data used in the script are available in supplementary data 14, Supplementary Material online.

## Supplementary Material

Supplementary data are available at *Genome Biology and Evolution* online.

## Supplementary Material

evab230_Supplementary_Data
