# Supplementary material for: Clustering of Sex-Biased Genes and Transposable Elements in the Genome of the Medaka Fish Oryzias latipes
Source: Genome Biol Evol. 2021 Oct 8;13(11):evab230. doi: 10.1093/gbe/evab230 (PMC8633743; doi:10.1093/gbe/evab230)
Supplement: evab230_Supplementary_Data [file evab230_supplementary_data.pdf]

Supplementary information:

Gene expression analysis details

Read mapping

The alignment of all read samples on the reference genome was performed with Hisat2 version 2.1.0 (Kim et al. 2019) using following command line options: **hisat2-build $Genome $GenomeIndex** to build an index of the genome; **hisat2 -p 7 -k 2 --dta -x $GenomeIndex -1 $Sample_1.fq.gz -2 $Sample_2.fq.gz -S $Sample.sam** to align each sample on the reference genome. The **--dta** option is used as recommended in the *New tuxedo* pipeline to perform an assembly of the transcripts (Pertea et al. 2016). The **-k 2** option allows to report a maximum of 2 alignments per sequencing read. As we wanted to exclude potentially expressed TEs from the assembly, we discarded multimapped reads using the following command line: **samtools view -h -@ 7 -f 0x2 $Sample.bam |awk '{if(substr($1, 0, 1)=="@" || $5==60){print $0}}' >$Sample.sam**. The option **-f 0x2** extracts the properly aligned paired reads from the bam. The *awk* command also keeps the header section of the SAM file as well as the reads with a MAPQ=60. In Hisat2, a MAPQ=60 means that the read has been uniquely aligned regardless of the mismatches / indels number. The filtered SAM file was finally converted in BAM and sorted: **samtools view -h -@ 7 -b -S $Sample.sam >$Sample_filtered.bam && samtools sort -@ 7 $Sample_filtered.bam sorted.bam && mv sorted.bam $Sample_filtered.bam.**

Transcript assembly

We used *StringTie* (Pertea et al. 2015) to assemble the transcripts using the genomic coordinates of the aligned reads, for each sample: **stringtie -p 7 -o $Sample_assemb.gtf -j 2 $Sample_filtered.bam && echo "$Sample$_assemb.gtf" >> mergelist.txt**. The **-j** option specifies that at least 2 reads are needed to support an exon-exon junction and create a new transcript. This is thus more stringent than the default value of 1 used by *StringTie*. One assembly per sample was created; we used the following command line to merge these assemblies: **stringtie --merge -o merged.gtf mergelist.txt.**

Transcript expression quantification

StringTie (Pertea et al. 2015) was also used to quantify transcript expression in each sample: **stringtie -e -B -p 7 -G merged.gtf -o $Sample/$Sample.gtf $Sample_filtered.bam**. **-e** option is used to estimate the expression of transcripts of the GTF file given to the **-G** option. **-B** option is used to create an output needed to run *ballgown* (Frazee et al. 2015) later.

Count matrix generation

We used the **prepDE.py** (https://github.com/gpertea/stringtie/blob/master/prepDE.py) script from *StringTie* (Pertea et al. 2015) to generate the count matrix from the output of the previous step.

Gene expression level quantification

We used the *ballgown* **R** package (Frazee et al. 2015) to estimate the TPM (Transcript Per Million) expression of each gene or transcript. Data were loaded with *ballgown* (Frazee et al. 2015) from the output of **StringTie -e -B**. Transcripts with low expression were filtered out as recommended in the *new tuxedo* procedure (Pertea et al. 2016) using: **subset(data,"rowMeans(texpr(data))>0.5", genomesubset=TRUE)** (supp. fig 1)**.** FPKM were then calculated using **texpr** function from *ballgown* (Frazee et al. 2015). TPM (Pachter 2011; Wagner et al. 2012) were finally computed from this table by normalizing the sum of each sample to 1e6.

Comparison between the new annotation and the reference gene annotation

Genes and transcripts reconstructed by *StringTie* (Pertea et al. 2015) were compared to the reference gene annotation of the genome using *gffcompare* (https://ccb.jhu.edu/software/stringtie/gffcompare.shtml). Each new transcript was assigned a class code depending on its similarity to a reference transcript, as described in *gffcompare* documentation. With this step we are able to know if each transcript found in our data is already present in the reference or if it is new.

Filtering of assembled transcripts

We applied different filters to get a clean set of coding or non-coding, expressed, and non-TE transcripts. Among the 45,444 transcripts, we selected those with a mean expression level higher than 0.5 FPKM (Fragment Per Kilobase per Million), leading to 38,033 transcript sequences. We then removed TEs from

transcripts as we wanted to analyze their expression with specialized tools. We ran RepeatMasker on the transcripts and generated the distribution of the percentage of the transcript masked by our bank (supp. fig. 18: Distribution of TE coverage of transcripts with a TE hit; supp. fig. 19: Distribution of TE coverage for all transcripts). We obtained a bimodal distribution suggesting that either the transcript corresponds to a TE or not. According to this distribution we set a threshold and discarded transcripts when more than 40% of their sequence was masked by our bank (35,213/38,033 transcripts kept making 20,588 genes; supp. fig. 17). Finally, we separated coding from non-coding transcripts with an ORF detection, to keep only transcripts having an ORF of more than 300 nucleotides, as recommended by *transdecoder* to be stringent enough (31,348/38,033 transcript with an ORF making 17,925 genes). Selecting genes with at least one coding transcript and considered as non-TE, we ended up with 17,254 coding genes and 3,334 non-coding genes (supp. fig. 17).

Differential expression analysis

The count tables of both genes and transcripts were generated using *prepDE.py* (see count matrix generation). The tables were then analyzed using the R package *DESeq2* (Love et al. 2014). We discarded the rows with less than 1 count to limit the number of multiple testing during the FDR correction. We used the *lfcShrink* function to shrink the fold change of genes with low counts. The method used was *apeglm* (Zhu et al. 2019) and we used a threshold of log2FC=1. It computes False Sign Or Small (FSOS, which gives the estimated rate of *false sign* among genes with equal or smaller s-value, analogous of the FDR q-values when looking at 2 thresholds: here 1 and -1) s-values instead of adjusted p-values (Stephens 2017). See DESeq2 vignettes for details on s-values (http://www.bioconductor.org/packages/release/bioc/vignettes/DESeq2/inst/doc/DESeq2.Html#differential-expression-analysis). We considered genes as differentially expressed between testis and ovary when the s-values were lower than 0.005 (FSOS = 0.5%). As the maximum FSOS is 50%, it is the analogous value for a FDR of 1%.

Correlation between TE and genes proximity, links between gene clusters and TE expression

The scripts used to generate the violin plot (fig. 4, supp. fig. 13) are available on the gitlab (see below). First, we ran *Correlation_violin.py.* The input files are a bedfile of the genes selected (coding, non-coding or both), and a bedfile of the TEs. Then, we ran *Correlation_violin.R,* with as input the expression of genes and TEs (supp. data 15 and 16), along with the output of the previous script. Figure 4 was generated using the script *Violin_mosaic.R* from the gitlab (see below). On the mosaic plot, the sum of the black value (37,038) is not equal to the total number of TE copies (37,108): some copies were removed as they were overlapping different gene clusters.

Supplementary data:

Supplementary data 1: TE bank generated from *O. latipes* genome in FASTA format.
Supplementary data 2: TE annotation of *O. latipes* genome using the TE bank.
Supplementary data 3: Table of the most expressed TE families.
Supplementary data 4-7: *Gene clusters* profiles for all chromosomes using coding genes only, non coding genes only, all genes or transposable elements.
Supplementary data 8: Fasta file of the reference reverse transcriptase used to generate the phylogeny.
Supplementary data 9: Fasta file of the reference reverse transcriptase retrieved from NCBI.
Supplementary data 10: Reverse transcriptase alignment using TE consensi.
Supplementary data 11: Phylogenetic tree generated using reverse transcriptase alignement from TE consensi.
Supplementary data 12: Reverse transcriptase alignment using Gypsy TE copies.
Supplementary data 13: Phylogenetic tree generated using reverse transcriptase alignement from Gypsy TE copies.
Supplementary data 14: Number of sex-biased TE copies per TE family.
Supplementary data 15: Gene expression data used to generate the Maplot.
Supplementary data 16: TE copies expression data.

Supplementary table 1: Total number and size of detected gene clusters using all genes, coding genes only, or non-coding genes only.

		**All genes (coding + non coding)**	**Coding genes only**	**Non-coding genes only**
Number of testis-biased clusters detected		32	9	3
Number of ovary-biased clusters detected		18	10	2
Size of testis-biased clusters	**Total**	28.945Mb	9.053Mb	20.46Mb
	Mean	905kb	1,006Mb	6.82Mb
	Minimum	455kb	850kb	5.5Mb
	Maximum	1.96Mb	1.360Mb	7.7Mb
Size of ovary-biased clusters	**Total**	18.2Mb	10.46Mb	12.98Mb
	Mean	1.011Mb	1.046Mb	6.49Mb
	Minimum	700kb	850kb	4.4Mb
	Maximum	1.715Mb	1.658Mb	8.58Mb
Number of genes in testis-biased gene clusters	**Total**	828	205	129
	Mean	26	23	43
	Minimum	7	8	33
	Maximum	56	54	55
Number of genes in ovary-biased clusters	**Total**	457	228	43
	Mean	25	23	22
	Minimum	4	6	13
	Maximum	64	40	30

Supplementary table 2: Genes with sexual-related function found in male- and female-biased gene clusters.

**Bias**	**Gene**	**Function**	**Reference (doi)**

Male	dnah9	Dynein axonemal heavy chain 9. This gene encodes the heavy chain subunit of axonemal dynein, a large multi-subunit molecular motor. Axonemal dynein attaches to microtubules and hydrolyzes ATP to mediate the movement of cilia and flagella.	10.1007/s003359900202
Male	morn3	Membrane occupation and recognition nexus repeat containing 3. Regulator of spermatogenesis.	10.4103/1008-682X.138186
Male	nsmce1	Non-structural maintenance of chromosomes element 1. Meiotic chromosome segregation.	10.1093/dnares/dsaa019
Male	pdgfd	Platelet-derived growth factors D. Cell proliferation.	10.1210/er.2010-0004
Male	dync2h1	Cytoplasmic dynein 2 heavy chain 1. Functions in cilia biogenesis.	10.1016/S0378-1119(97)00417-4
Male	fzd4	Frizzled-4. Involved in adult spermatogenesis.	10.1095/biolreprod.112.105809
Male	numa1	Nuclear mitotic apparatus protein 1. Binding partner of BRAP2 in human testis.	10.1016/j.bbamcr.2013.05.015
Male	cfap54	Cilia And Flagella Associated Protein 54.	10.1091/mbc.E15-02-0121
Male	dnajc18	DnaJ homolog subfamily C member 18. Might play a role during germ cell maturation in adult rat testis.	10.12717/DR.2017.21.3.237
Male	dnajb13	DnaJ homolog subfamily B member 13. Plays a role in the formation of the central complex of ciliary and flagellar axonemes.	10.1016/j.ajhg.2016.06.022
Male	ucp2	Mitochondrial uncoupling protein 2. Regulation of human spermatozoa motility.	10.1159/000494479
Male	armc3	Armadillo repeat-containing protein 3.	10.1186/s12863-016-0356-7
Male	cfap221	Cilia- and flagella-associated protein 221. May play a role in cilium morphogenesis.	10.1128/MCB.00354-07
Male	ccnb1ip1	E3 ubiquitin-protein ligase CCNB1IP1. Limiting factor for crossing-over during meiosis.	10.1128/MCB.23.6.2109-2122.2003
Male	lrguk	Leucine-rich repeat and guanylate kinase domain-containing protein. Involved in multiple aspects of sperm assembly including acrosome attachment, shaping of the sperm head and in the early aspects of axoneme development.	10.1371/journal.pgen.1005090
Male	ythdc2	3'-5' RNA helicase YTHDC2. Plays a key role in the male and female germline by promoting transition from mitotic to meiotic divisions in stem cells.	10.1371/journal.pgen.1006704
Male	cdkn2c	Cyclin-dependent kinase 4 inhibitor C.	
Male	cep350	Centrosome-associated protein 350. Required for ciliation.	10.1098/rsob.170114
Male	tdrd12	Putative ATP-dependent RNA helicase TDRD12. Probable ATP-binding RNA helicase required during spermatogenesis to repress transposable elements and preventing their mobilization, which is essential for the germline integrity.	10.1073/pnas.1316316110
Male	lamb1	Laminin subunit beta-1. Expressed in the developing male and female gonads and mesonephros.	10.1046/j.1432-0436.1997.6230129.x
Male	dmrt1a	Doublesex- and mab-3-related transcription factor 1A. Transcription factor that plays a key role in male sex determination and differentiation by controlling testis development and germ cell proliferation.	10.1242/dev.048751
Female	zan	Zonadhesin. Binds in a species-specific manner to the zona pellucida of the egg. May be involved in gamete recognition and/or signaling.	10.1086/508473
Female	bokb	Bcl-2-related ovarian killer protein homolog B. May play a role in apoptosis.	10.1038/sj.cdd.4402016
Female	buc	Bucky ball. Prion-like protein required for the formation of Balbiani body (electron-dense aggregates in the oocyte) and germ plasm assembly, and for the establishment of oocyte polarity during early oogenesis.	10.1016/j.ydbio.2008.05.557
Female	hsd17b1	Estradiol 17-beta-dehydrogenase 1. Favors the reduction of estrogens and androgens.	10.1096/fj.02-0026fje

Supplementary figure 1: MAPlot of coding gene expression in the gonads of *O. latipes*. Each dot represents a coding gene. The x-axis corresponds to the signal intensity averaged across all replicates, and the y-axis to the log2FC of expression between testis and ovary. The higher the log2FC of a coding gene is, the more it is over-expressed in testes (in blue, significantly, 3,600 genes), and the lower it is, the more it is over-expressed in ovaries (in red, significantly, 3,293 genes). The more the gene is on the right, the more it is overall expressed across all replicates. In green are displayed genes described in the literature as being involved in medaka sexual development and function (Nakamoto et al. 2006; Herpin et al. 2013; Horie et al. 2016; Kobayashi et al. 2017).

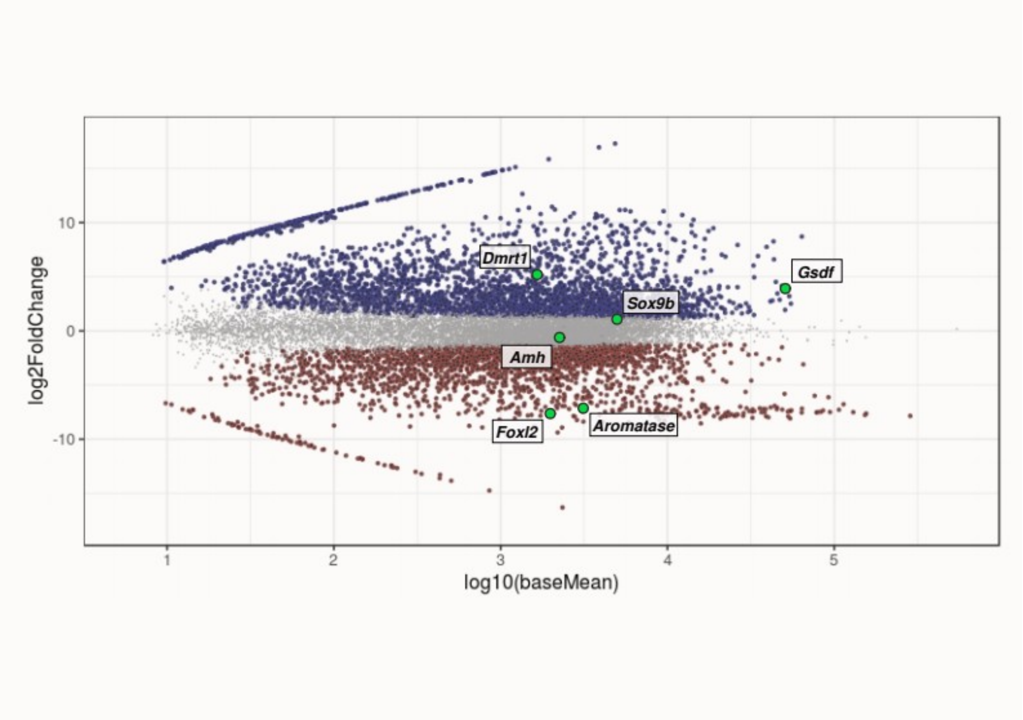

Supplementary figure 2: Number of sex-biased, expressed (but non-biased) and non-expressed TE copies in TE families enriched in sex-biased copies.

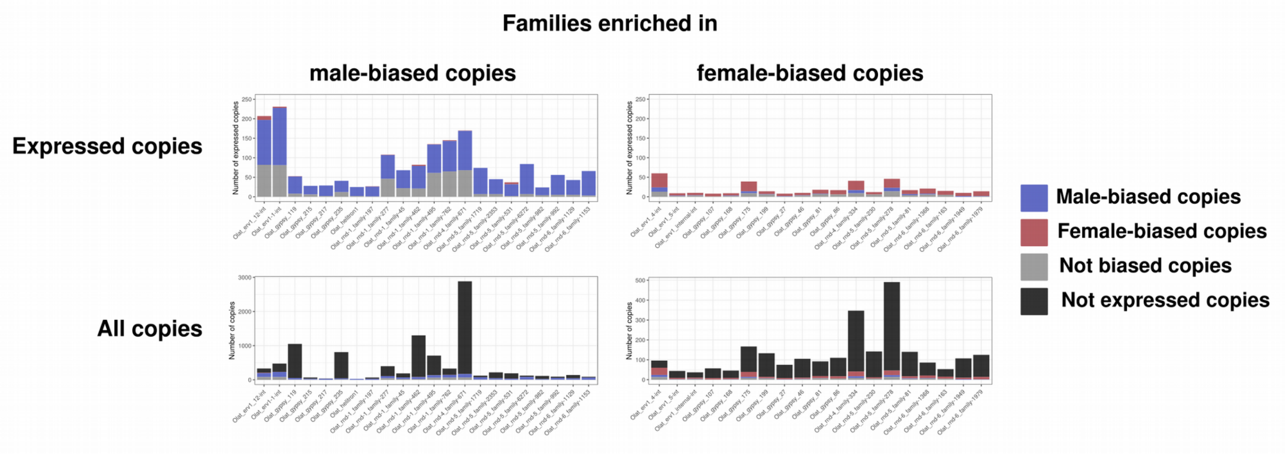

Supplementary figure 3: Phylogeny generated using the consensus of each LTR retrotransposon family.

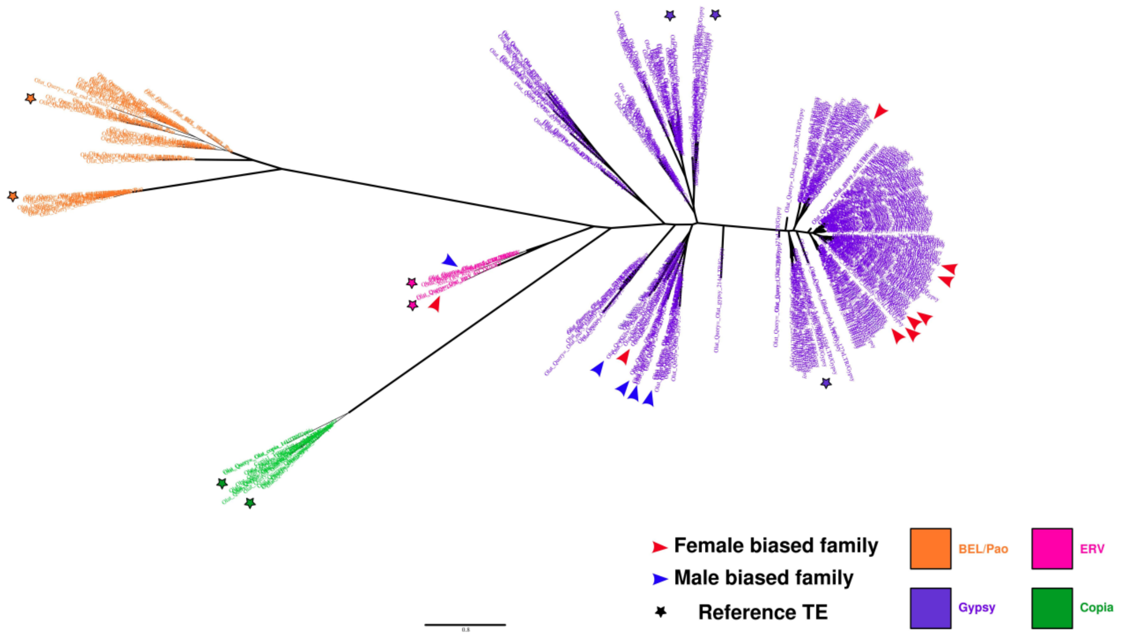

Supplementary figure 4: A. Phylogeny of expressed Gypsy TE copies of medaka using the amino acid sequence of the reverse transcriptase (RT). Tip colors correspond to the expression bias (red: ovary; blue: testis; black: expressed but not sex-biased). Four subtrees are highlighted (SUB1-4) for which we analyzed copy insertion sites. B. Distribution of GC % in 5kb upstream and downstream Gypsy insertion regions, for insertions of subtrees SUB1-4 from figure R3. GC% are not significantly different between subtrees (p-val = 0.790, ANOVA1). Blue: clusters of mainly male-biased copies; red: cluster of mainly female-biased copies; black: cluster of mainly non-biased copies.

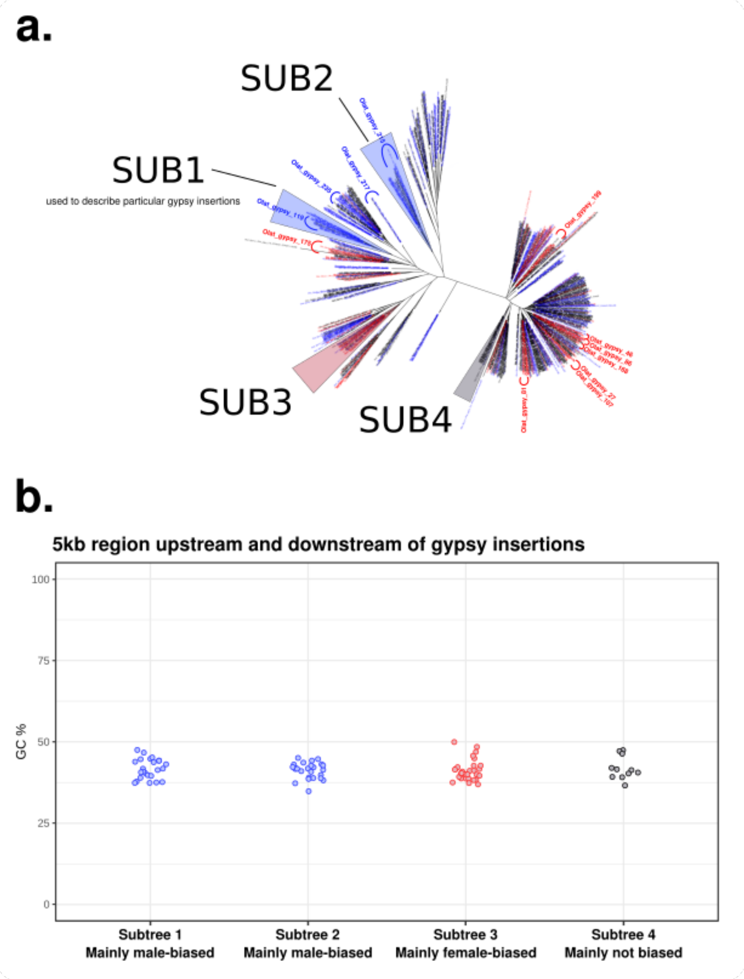

Supplementary figure 5: A. Structure of the 10 longest Gypsy insertions of the male-biased subtree 1 (fig. R3). Green boxes : Open Reading Frames ; yellow arrows : LTRs ; AP : Aspartic Protease ; RT : Reverse Transcriptase ; RH : RNAse H ; INT : Integrase. DE: Differentially Expressed. **B.** Phylogeny of expressed and non-expressed Gypsy TE copies of medaka using the amino acid sequence of the reverse transcriptase (RT). Colors correspond to the expression bias (red: ovary; blue: testis; green: expressed but not sex-biased; black: not expressed in gonads).

A

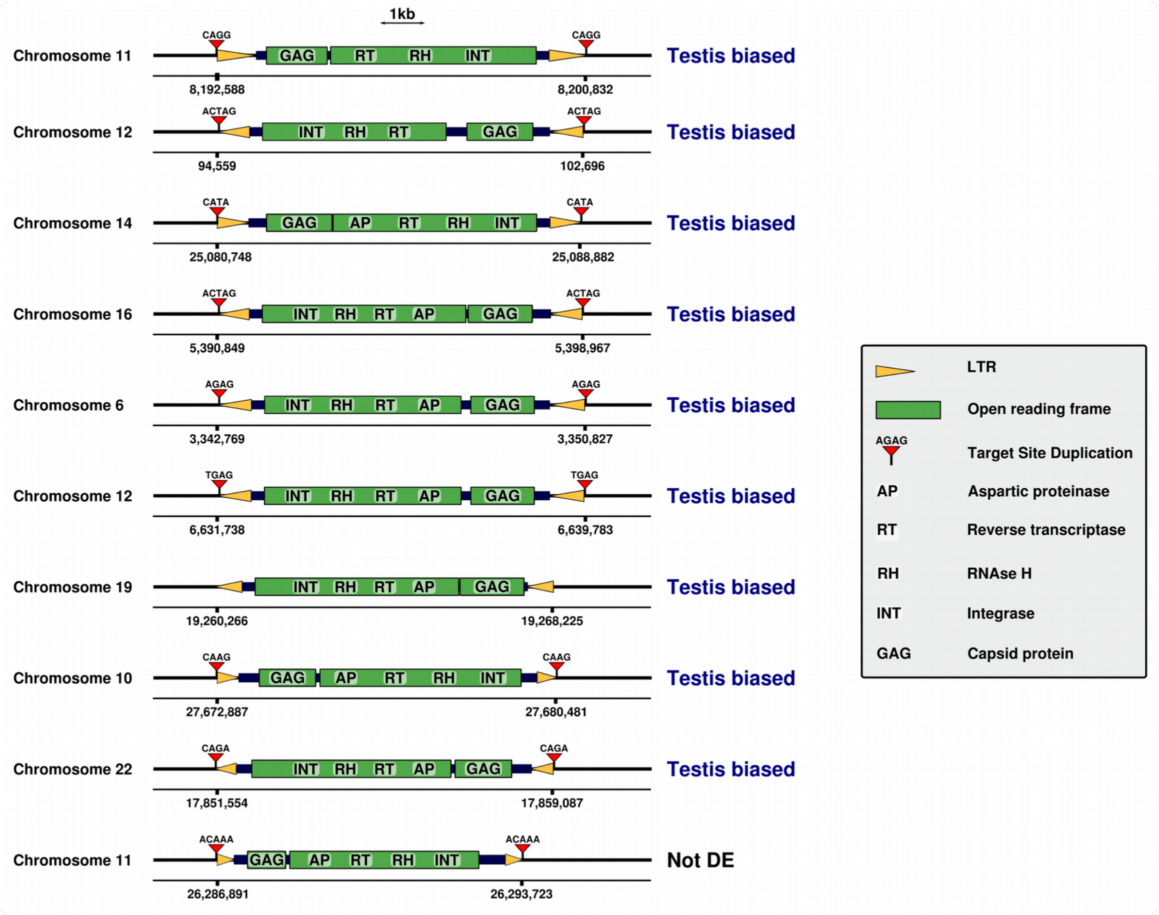

B

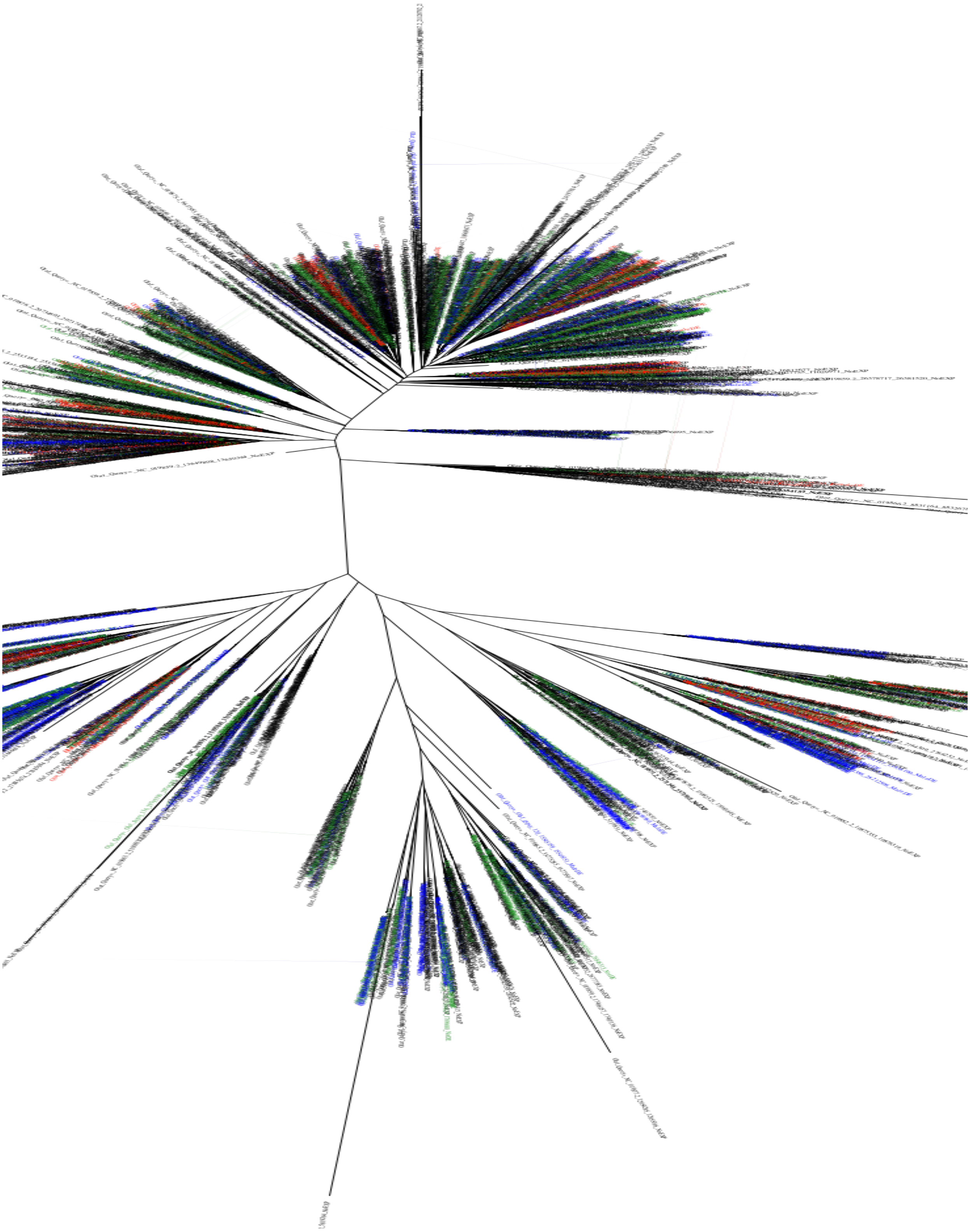

Supplementary figure 6: Stretches of genes obtained using only male-biased or female-biased coding genes or non-coding genes.

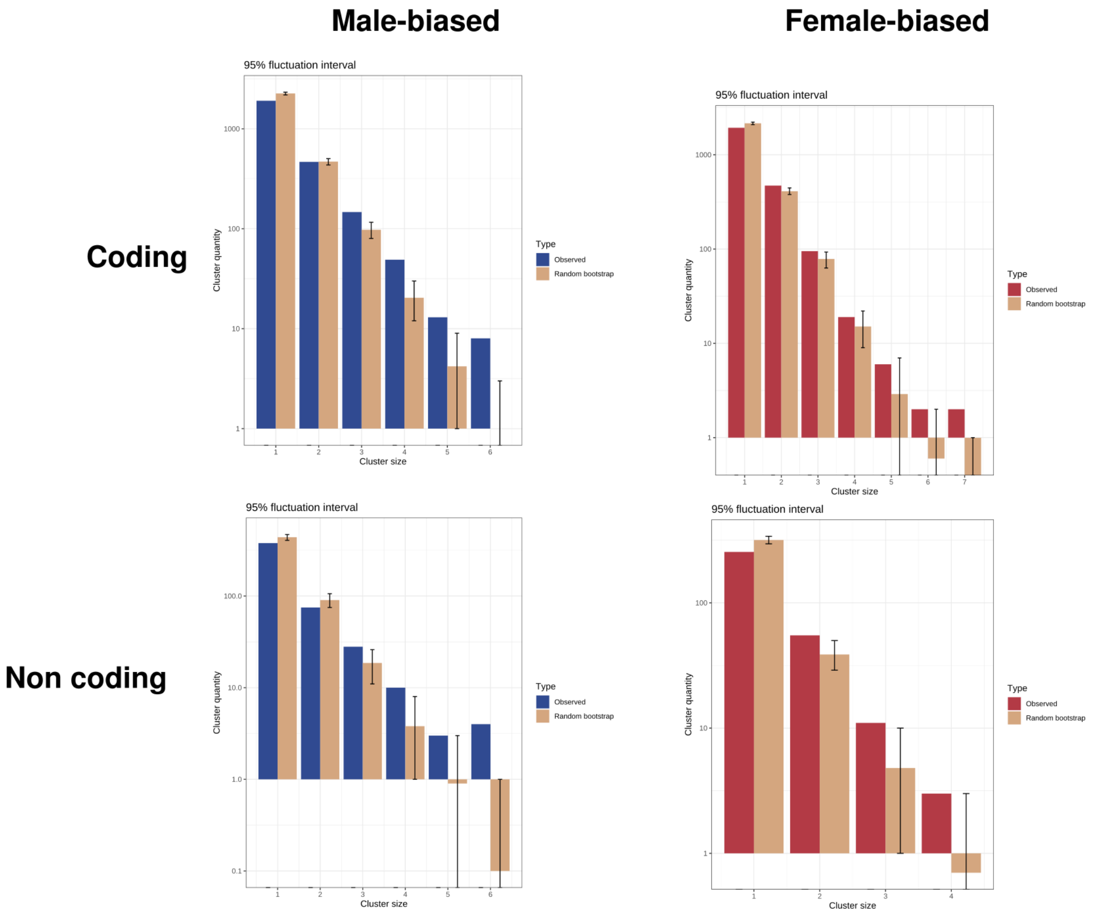

Supplementary figure 7: Representation of some clusters.
A. Male-biased gene cluster located on chromosome 4. This cluster contains numerous TEs that are particularly concentrated in the central region, both in intronic and intergenic regions.

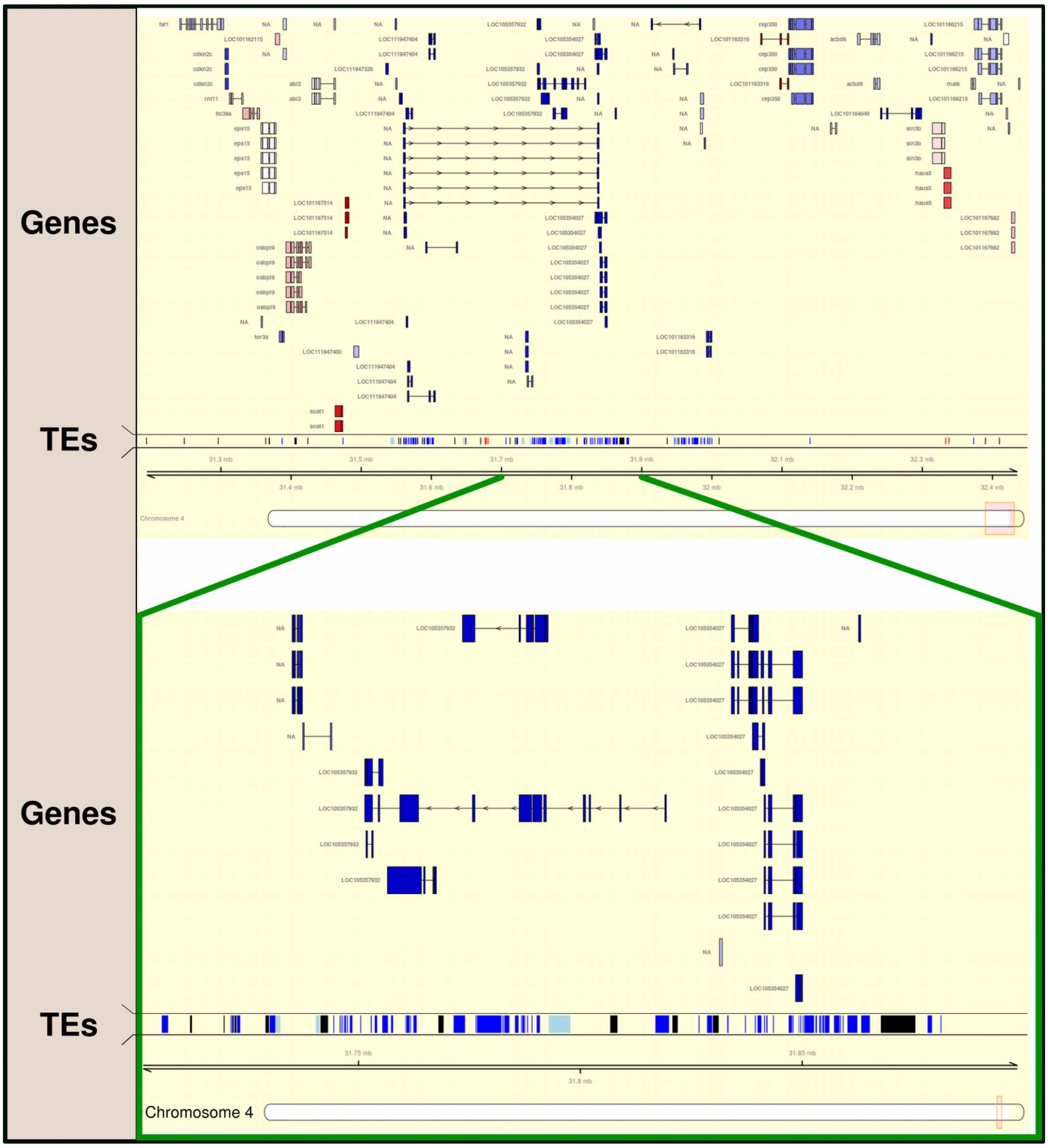

B. Female-biased gene cluster located on chromosome 15:

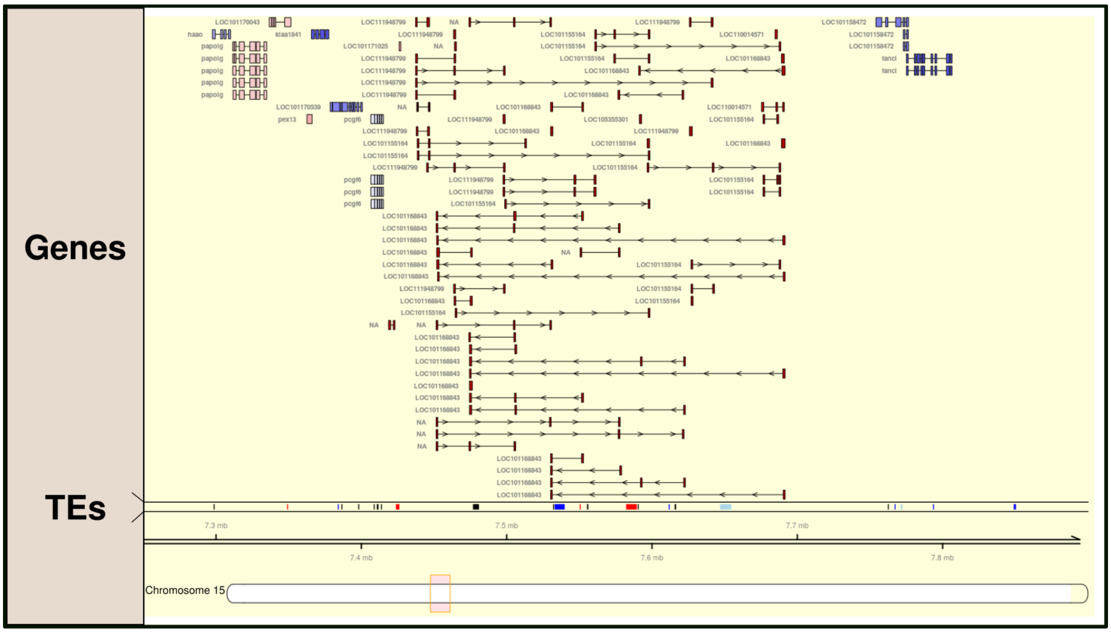

Supplementary figure 8: GO term enrichment of male-biased genes (P-val < 0.01). The red line indicates the proportion of GO terms expected by chance. The x-axis shows the observed proportion, with higher proportions on the right. Any proportion at the right of the red line is higher than expected by chance. The size of the points is proportional to the number of genes associated with the annotation. The color of the point indicates the associated P-value, with the lowest P-values in red. Most significant GO terms are at the bottom. Terms highlighted in beige are related to male sexual function.

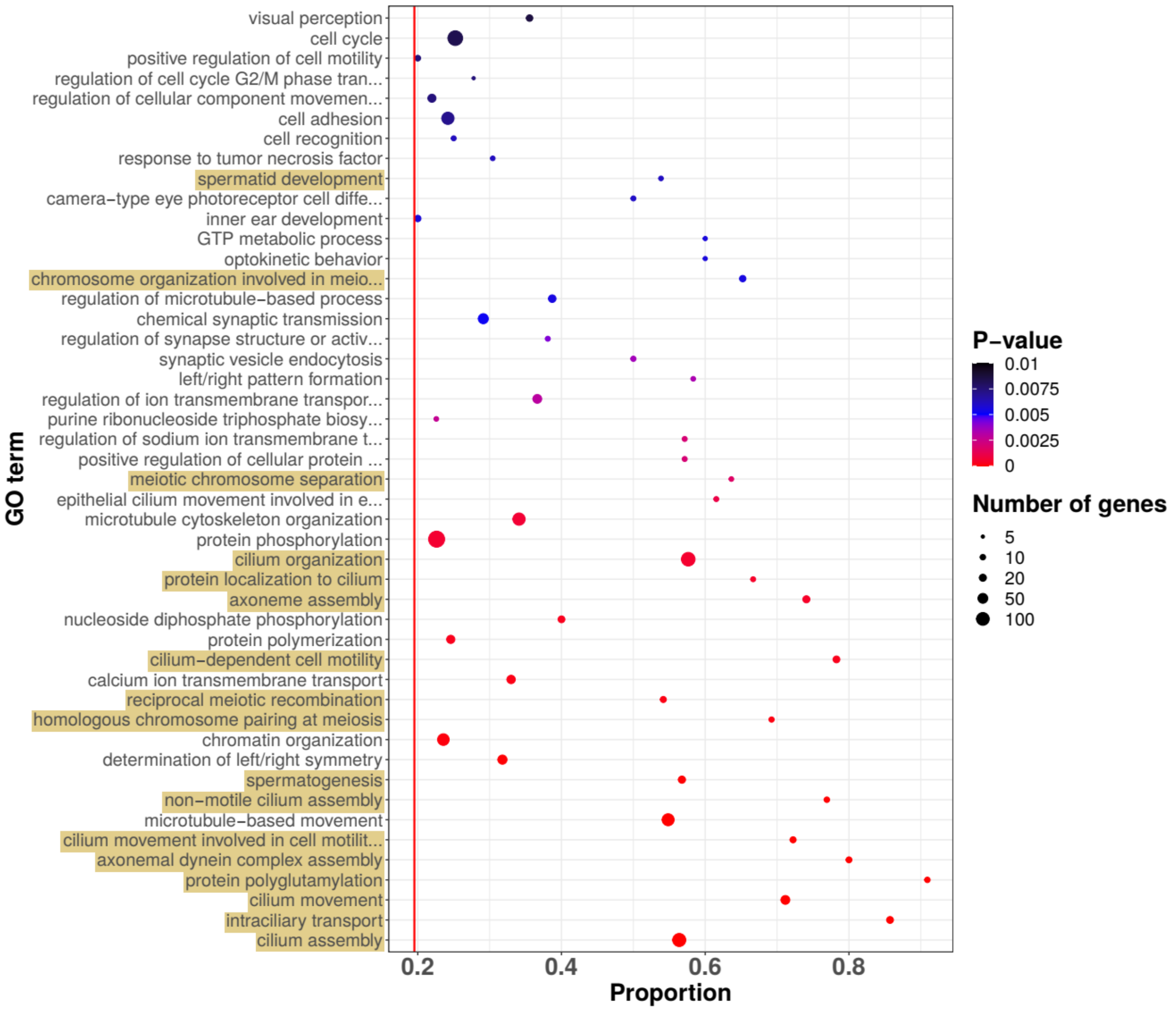

Supplementary figure 9: GO term enrichment of female-biased genes (P-val < 0.01). The red line indicates the proportion of GO terms expected by chance. The x-axis shows the observed proportion, with higher proportions on the right. Any proportion at the right of the red line is higher than expected by chance. The size of the points is proportional to the number of genes associated with the annotation. The color of the point indicates the associated P-value, with the lowest P-values in red. Most significant GO terms are at the bottom. Terms highlighted in beige are related to female sexual function.

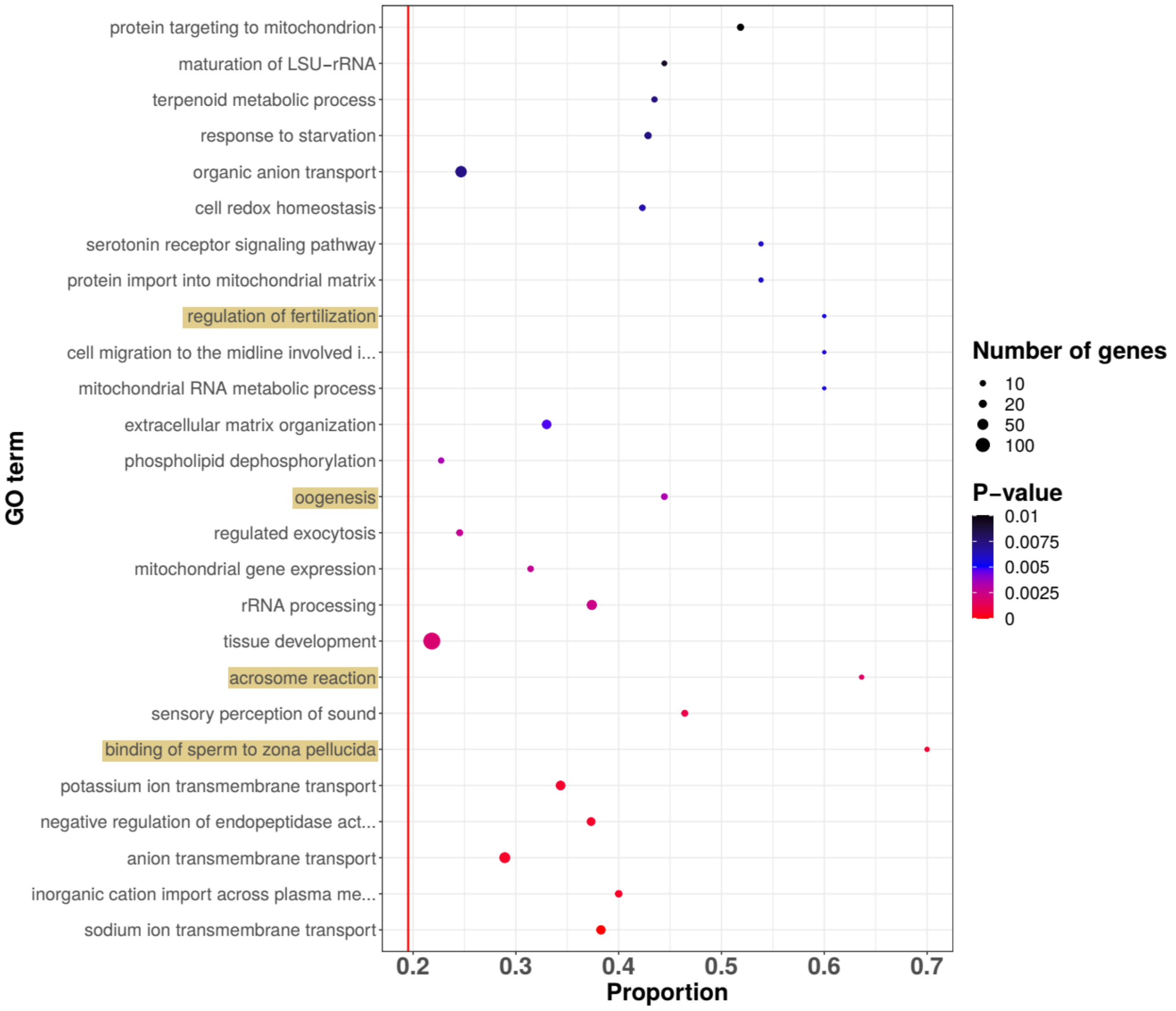

Supplementary figure 10: GO term analysis of genes in male-biased clusters (P-val < 0.05). The term 'spermatogenesis' is significantly found associated to these genes. Apart from this term, the other enrichments do not specifically refer to gonadal function. The red line indicates the proportion of GO terms expected by chance. The x-axis shows the observed proportion, with higher proportions on the right. Any proportion at the right of the red line is higher than expected by chance. The size of the points is proportional to the number of genes associated with the annotation. The color of the point indicates the associated P-

value, with the lowest P-values in red. Most significant GO terms are at the bottom. Terms highlighted in beige are related to male sexual function.

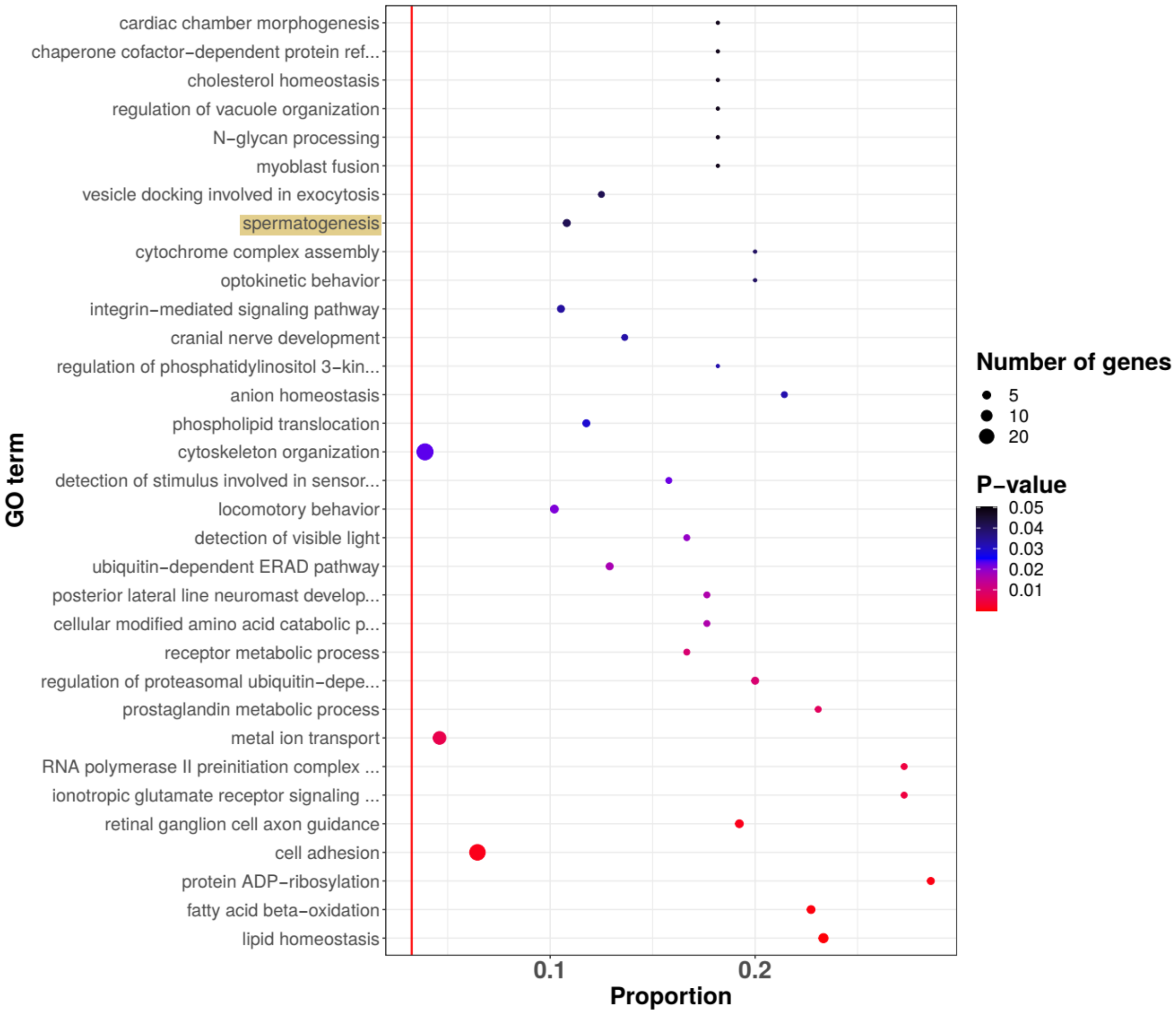

Supplementary figure 11: GO term analysis of genes in female-biased clusters (P-val < 0.05). No function obviously linked with female sexual function was found as enriched here. The red line indicates the proportion of GO terms expected by chance. The x-axis shows the observed proportion, with higher proportions on the right. Any proportion at the right of the red line is higher than expected by chance. The size of the points is proportional to the number of genes associated with the annotation. The color of the point indicates the associated P-value, with the lowest P-values in red. Most significant GO terms are at the bottom.

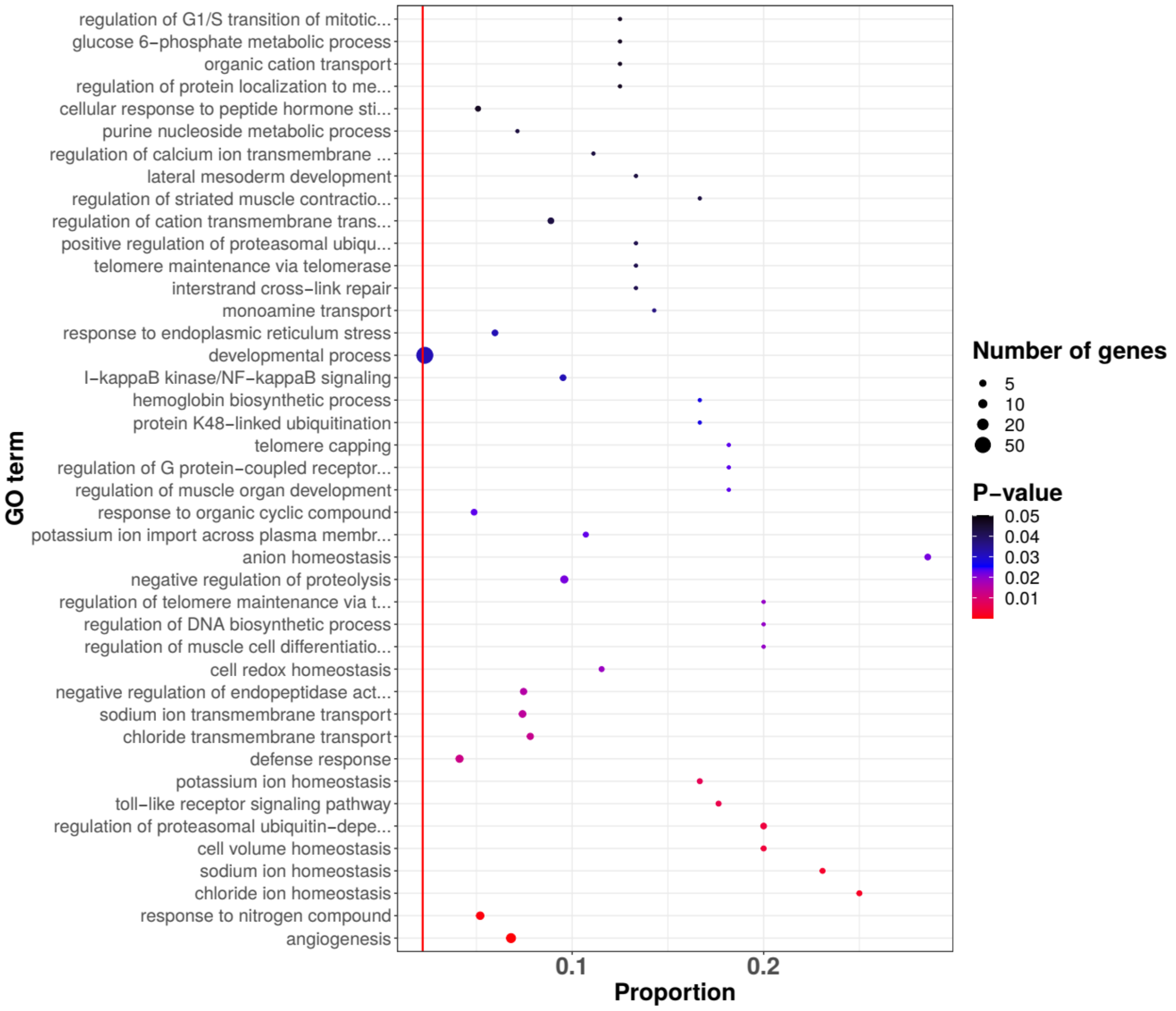

Supplementary figure 12: Genomic organization of the X chromosome region surrounding the Y-specific insertion carrying the Dmrt1bY master sex-determining gene of O. latipes. The region that was duplicated on chromosome Y concomitantly with the insertion of the Y-specific region is framed in pink. A cluster of male-biased TEs is located close to the duplicated region on both X and Y chromosomes. The color code reflects the expression bias (blue: male-biased, red: female-biased).

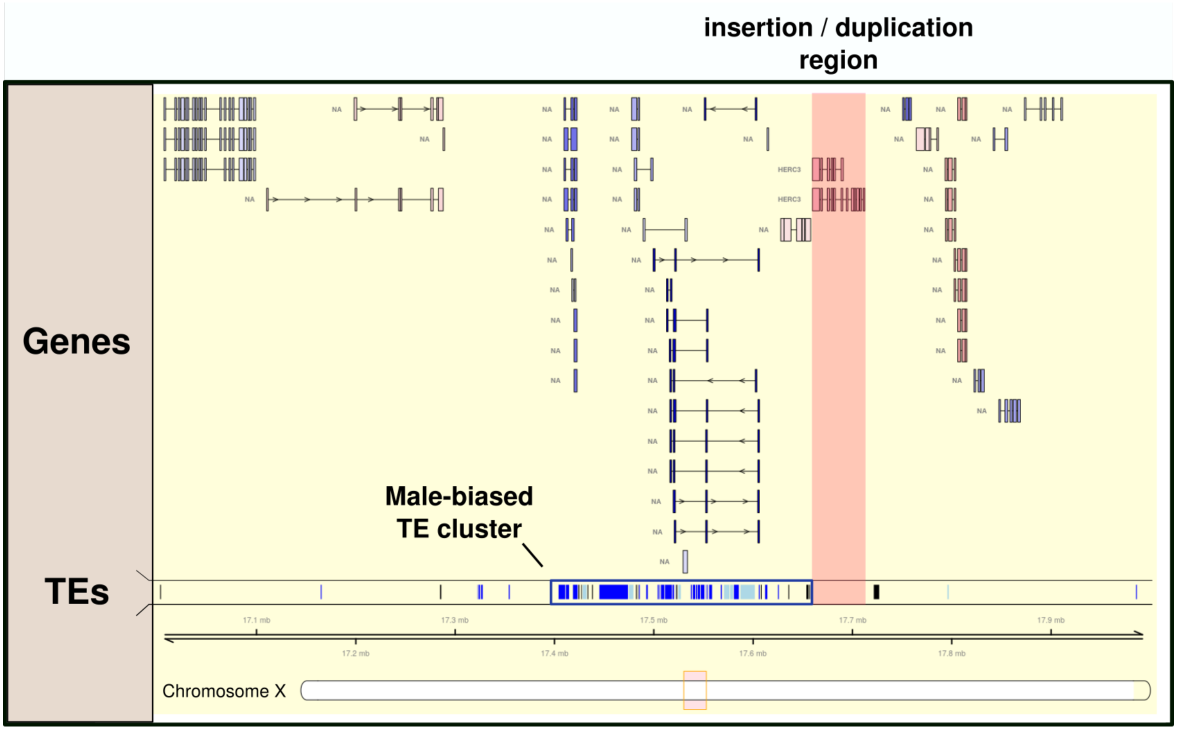

Supplementary figure 13: Gene-TE expression correlation using coding or non-coding genes only.

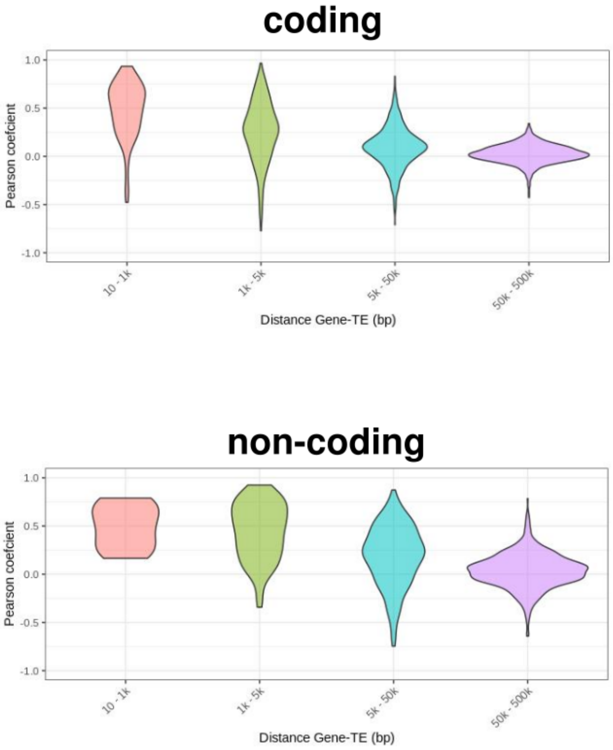

Supplementary figure 14: Sex-biased TE expression is dependent from the genomic location of sex-biased gene clusters. Expression differential of TE copies depending on their location in the genome, using clusters made from coding genes or non-coding genes only, or from both coding and non-coding genes.

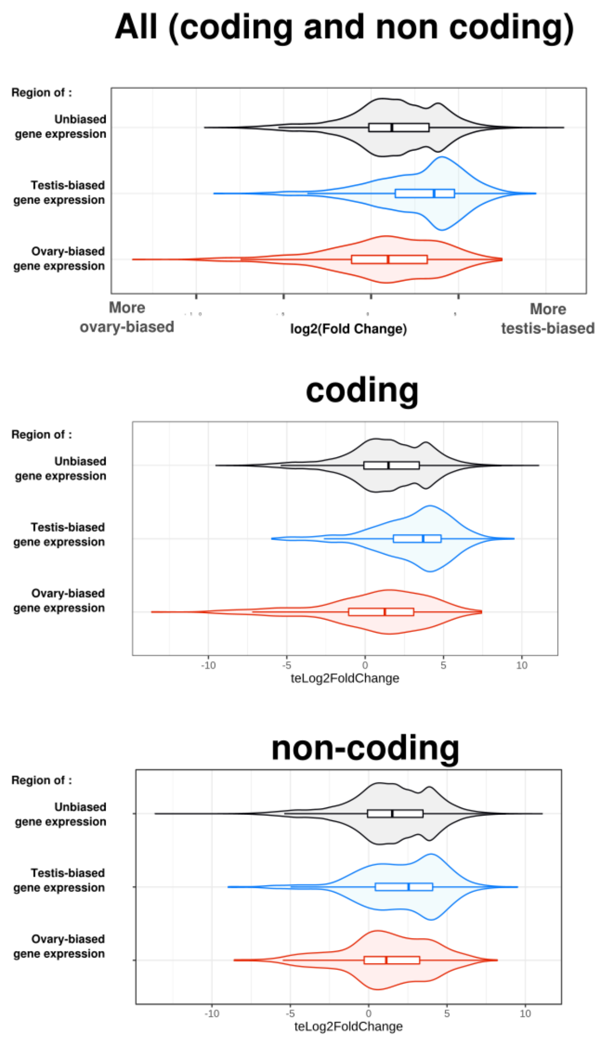

Supplementary figure 15: Mosaic plot representing the number of observed sex-biased vs. non-biased TE copies being located in sex-biased vs non-biased regions (as assessed by gene expression). The surface of each rectangle corresponds to the number of TE copies in each type of region, indicated in black (observed). The width of the rectangles corresponds to the proportion of copies located in the different types of regions, while the height of the rectangles corresponds to the proportion of testis- vs. ovary-biased vs. unbiased

copies located in each type of region. The expected number of copies calculated if there was no association between region and TE expression is indicated in grey (expected). When the number of copies is significantly higher than expected by chance (ratio > 1), the category is filled in green; when it is significantly lower, the category is filled in purple (ratio <1). As some copies are present in overlapping sex-biased regions, we selected the 37,038 copies unambiguously associated to a region (sum of the black values).

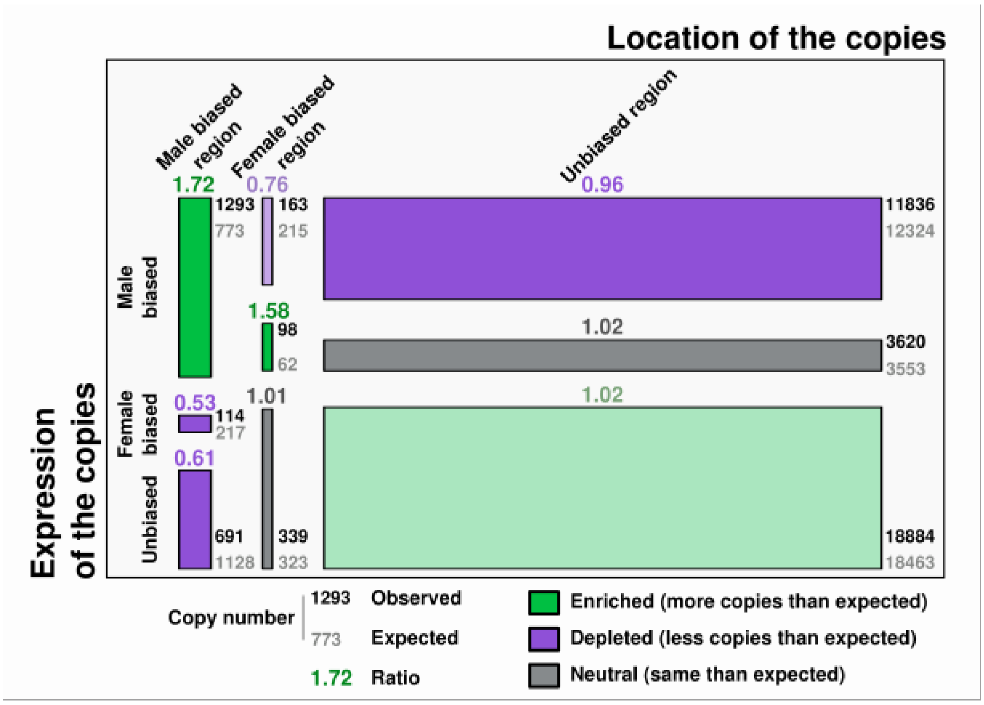

Supplementary figure 16: rnd-5_family-992 TE copies alignment and localization of the predicted SOX8 and HOXD13 binding sites. Copies localized in male-biased clusters in O. latipes are framed in yellow.

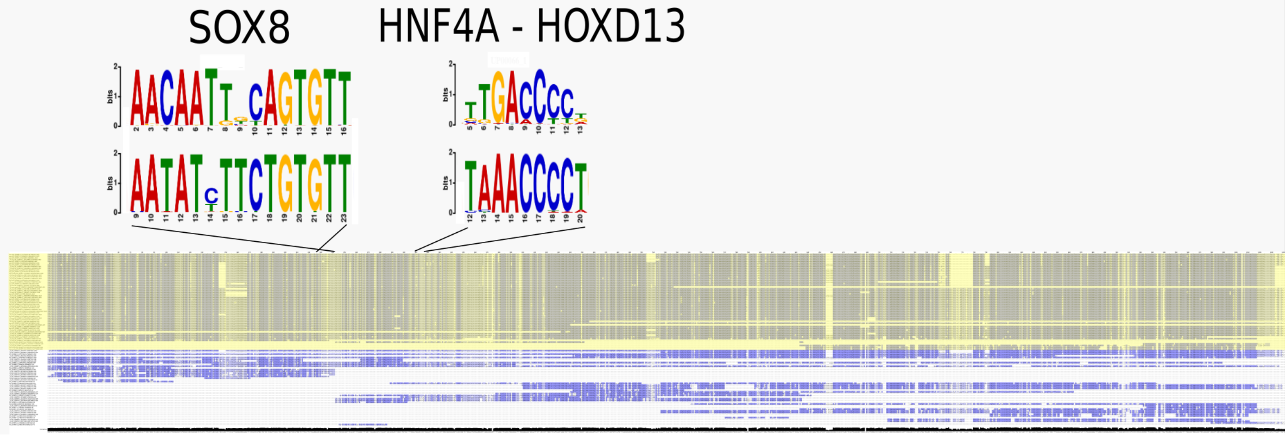

Supplementary figure 17: Venn diagram showing the number of transcripts and genes considered as coding and non-coding. A coding gene has at least one coding transcript with an ORF longer than 300 nucleotides. A non-coding gene has only non-coding transcripts.

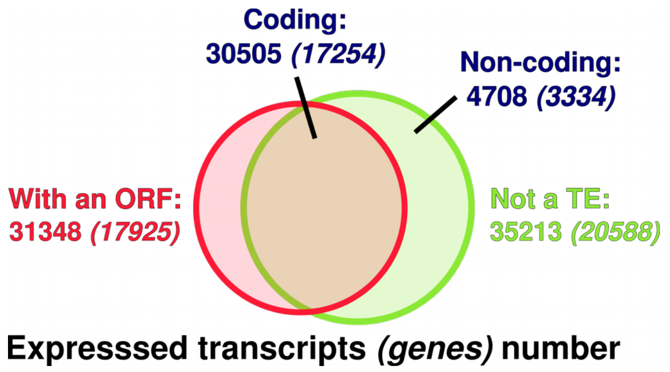

Supplementary figure 18: Percentage of the transcripts covered by a TE (% length) after removing transcripts that do not overlap a TE.

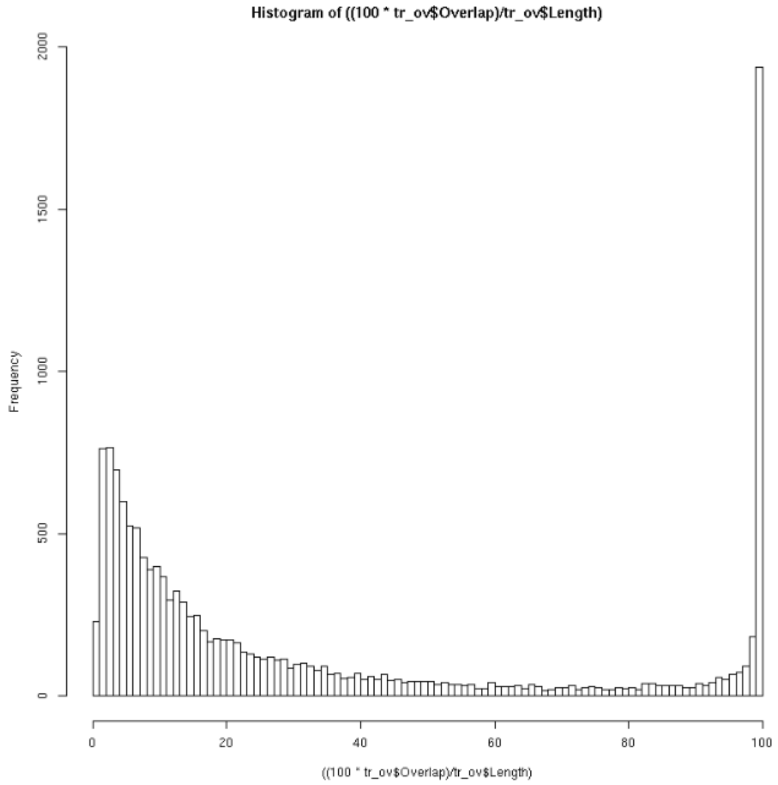

.

Supplementary figure 19: Percentage of the transcripts covered by a TE (% length) using all transcripts.

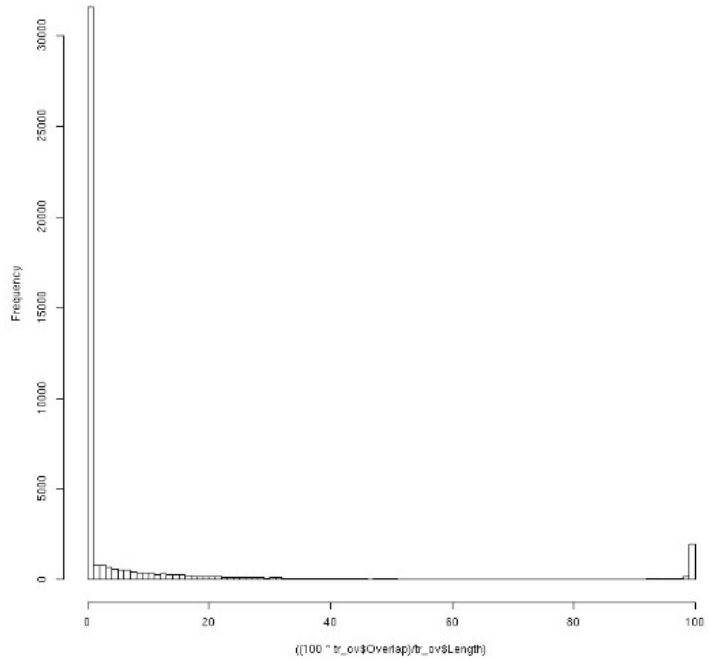

Frazee AC, Pertea G, Jaffe AE, Langmead B, Salzberg SL, Leek JT. 2015. Ballgown bridges the gap between transcriptome assembly and expression analysis. Nature Biotechnology. 33:243–246.

Herpin A, Adolfi MC, Nicol B, Hinzmann M, Schmidt C, Klughammer J, Engel M, Tanaka M, Guiguen Y, Schartl M. 2013. Divergent expression regulation of gonad development genes in medaka shows incomplete conservation of the downstream regulatory network of vertebrate sex determination. Molecular Biology and Evolution. 30(10):2328–2346.

Horie Y, Myosho T, Sato T, Sakaizumi M, Hamaguchi S, Kobayashi T. 2016. Androgen induces gonadal soma-derived factor, Gsdf, in XX gonads correlated to sex-reversal but not Dmrt1 directly, in the teleost fish, northern medaka (Oryzias sakaizumii). Molecular and Cellular Endocrinology. 436:141–149.

Kim D, Paggi JM, Park C, Bennett C, Salzberg SL. 2019. Graph-based genome alignment and genotyping with HISAT2 and HISAT-genotype. Nat Biotechnol. 37(8):907–915.

Kobayashi T, Myosho T, Yamamoto J, Okamura T, Onishi Y, Sakaizumi M, Hamaguchi S, Iguchi T, Horie Y. 2017. Estrogen alters gonadal soma-derived factor (Gsdf)/Foxl2 expression levels in the testes associated with testis-ova differentiation in adult medaka, Oryzias latipes. 191:209–218.

Love MI, Huber W, Anders S. 2014. Moderated estimation of fold change and dispersion for RNA-seq data with DESeq2. Genome Biology. 15(12):550.

Nakamoto M, Matsuda M, Wang D-S, Nagahama Y, Shibata N. 2006. Molecular cloning and analysis of gonadal expression of Foxl2 in the medaka, Oryzias latipes. Biochemical and Biophysical Research Communications. 344(1):353–361.

Pachter L. 2011 May 12. Models for transcript quantification from RNA-Seq. arXiv:11043889 [q-bioGN].

Pertea M, Kim D, Pertea GM, Leek JT, Salzberg SL. 2016. Transcript-level expression analysis of RNA-seq experiments with HISAT, StringTie and Ballgown. Nature Protocols. 11(9):1650–1667.

Pertea M, Pertea GM, Antonescu CM, Chang T-C, Mendell JT, Salzberg SL. 2015. StringTie enables improved reconstruction of a transcriptome from RNA-seq reads. Nature Biotechnology. 33(3):290–295.

Stephens M. 2017. False discovery rates: a new deal. Biostatistics. 18(2):275–294.

Wagner GP, Kin K, Lynch VJ. 2012. Measurement of mRNA abundance using RNA-seq data: RPKM measure is inconsistent among samples. Theory Biosci. 131(4):281–285.

Zhu A, Ibrahim JG, Love MI. 2019. Heavy-tailed prior distributions for sequence count data: removing the noise and preserving large differences. Bioinformatics. 35(12):2084–2092.
